# First foods: Diet quality among infants aged 6–23 months in 42 countries

**DOI:** 10.1016/j.foodpol.2019.101762

**Published:** 2019-10

**Authors:** Samira Choudhury, Derek D. Headey, William A. Masters

**Affiliations:** a Centre for Development, Environment and Policy, School of Oriental & African Studies, London WC1H 0XG, UK; b Poverty, Health and Nutrition Division, The International Food Policy Research Institute (IFPRI), Washington, DC, USA; c Friedman School of Nutrition Science & Policy, Tufts University, Boston, MA, USA

**Keywords:** Child malnutrition, Child diets, Dietary diversity, Bennett’s law

## Abstract

Diet quality is closely linked to child growth and development, especially among infants aged 6–23 months who need to complement breastmilk with the gradual introduction of nutrient-rich solid foods. This paper links Demographic and Health Survey data on infant feeding to household and environmental factors for 76,641 children in 42 low- and middle-income countries surveyed in 2006–2013, providing novel stylized facts about diets in early childhood. Multivariate regressions examine the associations of household socioeconomic characteristics and community level indicators of climate and infrastructure with dietary diversity scores (DDS). Results show strong support for an infant-feeding version of Bennett's Law, as wealthier households introduce more diverse foods at earlier ages, with additional positive effects of parental education, local infrastructure and more temperate agro-climatic conditions. Associations with consumption of specific nutrient-dense foods are less consistent. Our findings imply that while income growth is indeed an important driver of diversification, there are strong grounds to also invest heavily in women’s education and food environments to improve diet quality, while addressing the impacts of climate change on livelihoods and food systems. These results reveal systematic patterns in how first foods vary across developing countries, pointing to new opportunities for research towards nutrition-smart policies to improve children’s diets.

## Highlights

• Nutrient-dense solid foods are needed to complement breastmilk after 6 months.
• Data from 76,641 infants reveals which food groups are used at each age.
• We link intake to parental education, household wealth, climate and infrastructure.
• Infant feeding follows Bennett's law, also mother's schooling and local conditions.
• Low diet diversity in early infancy calls for new actions to meet nutrient needs.

## 1 Introduction

Undernutrition limits the growth and development of several hundred million children every year, contributing to poor health and cognition, low educational attainment and reduced lifetime earnings (Glewwe et al., 2001, Horton and Alderman, 2008, Hoddinott, 2009). Growth faltering is most severe in the 6–23 month window, when infants are at high risk of inadequate nutrient intakes because the nutrient density of family foods is typically low in relation to the high energy and nutrient requirements needed to support rapid growth at this critical stage of development (Shrimpton et al., 2001, Victora et al., 2010). To prevent growth faltering, after 6 months of exclusive breastfeeding infants need nutrient-rich foods to complement continued breastfeeding and a gradual transition to the family diet after age two (PAHO/WHO, 2003).

In this study we explore why children 6–23 months are fed what they are fed in 42 low- and middle-income countries (LMICs), as measured through standard dietary diversity metrics and consumption of specific food groups in nationally-representative surveys. The scope of this study is novel, but also builds on existing literatures in nutrition and economics.

Nutritionists have long recognized the importance of gradually introducing new foods in addition to continued breastfeeding after 6 months of age (WHO/UNICEF 2003). Observations of actual food intake by month in LMICs is generally limited to small scale qualitative studies in specific communities (Pak-Gorstein et al., 2009), and population-representative survey data has typically been aggregated into wider age categories if only because each survey can reach only a few children at each age. Previous nutrition-focused studies have examined differences in child diets in specific countries (Dibley and Senarath, 2012, Begin, 2017, Galway et al., 2018) or intake of specific food groups (e.g. animal sourced foods) across countries (Headey et al., 2018a). This study focuses on general dietary diversity metrics as well as consumption of specific foods, cover geographical as well as household level predictors of diversification, and does so in a broad swathe of countries.

Economics also has a long tradition of research on consumer demand for variety in other kinds of goods, showing introduction of new goods and increased diversity in consumption as incomes rise (Senior, 1836, Marshall, 1890, Jackson, 1984). For food in particular, since Bennett (1941) many studies have confirmed that richer people with higher expenditure levels diversify out of starchy staples into more costly and more nutrient-dense foods, whether using national data on per-capita food supplies (e.g. Choudhury and Headey, 2017) or survey data (e.g. Behrman and Deolalikar, 1989, Subramanian and Deaton, 1996).

Recent studies show how the diet diversification process is affected by differences in the relative cost of acquiring different foods, including the many situations in which certain foods are entirely missing from local markets (De Janvry et al., 1991, Hoddinott et al., 2015). As shown by Singh et al. (1986), at times and places where people can buy and sell freely at market prices, consumption decisions would be separable from production choices as farm households would raise whatever crops and livestock would generate their highest possible standard of living. A key insight of this literature is that many nutrient-dense foods such as fruits and vegetables, meat, eggs and milk are not easily stored or traded in underdeveloped rural settings, so they can be included in child diets only when and where they are produced (e.g. Hoddinott et al., 2015). Studies of non-separability typically focus on whether the household's own production is needed for consumption, but trade among villagers could ensure that the more relevant agro-ecological constraint is at the community level. Even when communities are linked through year-round trade, price differentials due to transport and storage costs ensure that local conditions affect relative prices, with more nutritious foods available at lower relative cost at places and times with more favourable agroecological and infrastructural conditions (Masters et al., 2018, Headey and Alderman, 2019). Previous work on how local agriculture and the food environment influence diets in low-income countries has focused primarily on overall diet diversity for households relative to their own production diversity (Sibhatu and Qaim, 2018), with more limited research on specific foods and food groups consumed by children at different ages (Mulmi et al., 2017).

This paper aims to provide a comprehensive exploration of the relative roles of wealth, parental knowledge/education, women’s empowerment, and geographical characteristics such as agroecology and infrastructure in shaping infant feeding patterns. We use recent Demographic and Health Surveys (DHS) from 42 countries, combined with geographic data about the locations of DHS survey clusters as described in Section 2. We use these data to first document infant feeding patterns in our sample, including differences across major developing regions, before presenting econometric models that account for inter-child differences in dietary diversity scores, minimum dietary diversity, and the consumption of eight nutrient-rich food groups. We then provide various extensions to explore the relationship between household and community level factors, to account for regional heterogeneity, and to contrast our results to findings from the existing literature, with potential policy implications and areas for future research.

## 2 Theory, data and methods

Our work is motivated by household decisionmaking models such as those described in Hoddinott et al., 2015, Behrman and Deolalikar, 1989, and Singh et al. (1986). We expect that parents seek to sustain child growth and development through age-specific nutrient intake and non-food goods or services such as healthcare, while also pursuing other objectives against a variety of resource constraints. Child outcomes also depend on intrahousehold bargaining, as each household member makes different contributions and has different preferences (Strauss and Thomas, 1995, Behrman, 1997, Haddad et al., 1997).

In this framework, if there were perfectly competitive markets for everything, child dietary intake would depend only on the household's wealth and full income (including the value of time and things produced at home), their preferences (including knowledge, empowerment and bargaining power within the household), and their food environment (including the relative prices and convenience of obtaining different foods). Missing or imperfect markets ensure that the household's own production possibilities and local conditions around each survey site also influence consumption, especially for bulky and highly perishable foods that have high transport and storage costs. These include many of the most important nutrient-dense foods needed for infant feeding, such as dairy products, eggs and many fruits and vegetables. Crop choice and productivity is very sensitive to temperature and climate (Schlenker and Lobell, 2010), and temperature patterns can also affect vectors for human and livestock diseases such as the tsetse fly, which has long limited availability of dairy products in Africa (Alsan, 2015), as well as the choice of livestock breeds with associated impacts on productivity. In this study we link these local agroecological and also infrastructural factors to infant feeding practices, comparing community characteristics directly to household and other influences on the first foods consumed by infants as they grow.

### 2.1 Data

We use multi-country household survey data from Phases 5 and 6 of the DHS (ICF International, 2015), which we then combine with administrative data, satellite imagery and climatological information for each household location drawn from the Harvest Choice database (IFPRI, 2018). Since the mid-2000s (Phase 5) the DHS has implemented a dietary module as measured by a simple yes/no indicator in which mothers/caretakers are asked to recall which of 7 food groups the child (0–24 months of age) consumed in the last 24 h. The DHS are particularly useful for multi-country analysis due to their standardized methods for study design and interviewer training, as well as careful translation to elicit comparable information about local foods and feeding practices. We focus specifically on infants after 6 months, because the WHO and others recommend exclusive breastfeeding for children 0–5 months of age.

Our primary measure of diet quality is the dietary diversity score (DDS), defined as the number of food groups (out of seven in total) consumed by a child in the past 24 h. In addition to this diversity metric, we explore consumption patterns of four nutrient-rich vegetal foods and four nutrient-rich animal-sourced foods. The seven food groups included in the DDS measure are described in Table 1, along with the eight nutrient-rich food groups. By definition, these scores focus on food groups defined in terms of plant and animal species, and omit information on intake of processed or packaged foods such as premixed infant cereals whose nutritional content is uncertain (Masters et al., 2017).

**Table 1:** Food group classifications used for diet diversity measurement.

Food groups used for dietary diversity scores	Disaggregated food categories used for analysis of nutrient-rich food consumption
(1) Starchy staples	(1) *Cereals (excluded)* (2) *Roots/tubers (excluded)*
(2) Legumes and nuts	(3) Legumes and nuts
(3) Vitamin A-rich fruits and vegetables	(4) Vitamin A-rich fruits and vegetables, excluding DGLV (5) DGLV (Dark green leafy vegetables)
(4) Other fruits/vegetables;	(6) Other fruits and vegetables
(5) Dairy products	(7) Dairy products
(6) Eggs	(8) Eggs
(7) Meat, organs, fish.	(9) Meat/organs (10) Fish*

Note:

* Data on fish consumption are missing for Peru.

Overall, the dietary intake data we use are available for 76,641 children aged 6–23 months in 42 countries in five regions, thus providing substantial socioeconomic and geographic variation for our analysis (see Table A1 for a full list of countries): most (58%) of our sample is from sub-Saharan Africa, with 24% from Latin America and the Caribbean, 11% from Asia and 7% from the Middle East and North Africa).

The theoretical framework described above emphasizes the importance of budget constraints in shaping food purchases. To capture purchasing power, we use DHS data on the household's ownership of durable consumption goods (radio, TV, refrigerator, motorbike, car) and housing characteristics (floor materials, access to electricity, access to piped water or an improved toilet) that reflect permanent income. Time-varying purchasing power would be reflected in household expenditure, but it is measured with substantial error (Beegle et al., 2012) and asset-based measures are generally preferred. Following the standard approach we use durable consumption goods to derive a wealth index using principal components analysis, as per Filmer and Pritchett (2001), re-estimating the index over our dataset of 42 countries to derive an internally comparable index using common weights. This multi-country asset index is very highly correlated with separately estimated country-specific asset indexes (r = 0.97), indicating that these asset categories have similar associations with each other and a robust ability to predict the latent concept of households' permanent income, despite differences in relative cost and demand across rural and urban households in various countries (Rutstein et al., 2013). Appendix Table A7 reports the asset scores created by the principal components analysis.

In addition to wealth, we use several other socioeconomic indicators that we interpret primarily as proxies for knowledge and preferences. Formal education, in particular has been shown to be a strong predictor of nutritional knowledge (Webb and Block, 2004, Schneider and Masters, 2019). We measure formal education as years of schooling of mothers and their partners (usually the father of the child). Following Alderman and Headey (2018), we pool years of education into different year brackets to allow for non-linear returns to education, whereby 1–6 years approximates “primary education”, 7–9 years denote “lower secondary” or “middle school” and 10-plus refer to attending “upper secondary” or receiving some amount of tertiary education. It is also possible that exposure to health services may impart nutritional knowledge relevant to diets and feeding practices. To this end we construct a health access index that equals one if mothers had antenatal check-ups, neonatal care (a medical facility birth) and postnatal care in the form of vaccinations. These three indicators are correlated with each other, and robustness tests revealed that each had similar coefficients when entered separately into DDS regressions. We also use an indicator of whether a child was breastfed within one hour of birth, as recommended by nutritionists, as a proxy for exposure to nutrition-specific counselling. And in addition to knowledge proxies, we also use women’s participation in decisions on her own healthcare as a proxy for maternal empowerment, which may be particularly important insofar as mothers are usually directly responsible for feeding young children. We also include the sex of the child to assess whether there are gender differences in child feeding, given evidence of biases in breastfeeding in countries such as India (Jayachandran and Kuziemko, 2009). Robustness tests using a variety of other DHS data as control variables did not alter results and are not reported here.

Our selection of community-level GIS indicators is motivated by the microeconomic theory around missing markets described above. Access to markets through improved infrastructure or inherent geographical advantages may be an important prerequisite for purchasing a greater variety of foods, especially perishable foods that are costly to trade long distances. The DHS records whether a cluster is urban or not, but definitions of urban vary substantially across countries and are somewhat arbitrary. Therefore, we use GIS estimates of travel time to cities to construct a “remote location” dummy variable that equals one if the DHS cluster (“village”) has more than a one-hour travel time to a town/city of 20,000 people or more, with that threshold suggested by nonparametric evidence from the association between DDS and travel times. We also use a satellite-based night lights intensity index to capture local economic development and electricity infrastructure (Henderson et al., 2012), as well as distance to coastline (to reflect international costs of importing food), distance to a major inland water body (a 10 km^2^ lake or a major river) to reflect access to fisheries and large scale irrigation, and population density of the surrounding area to reflect the thickness of local markets.

In addition to infrastructural and demographic conditions, agricultural conditions can substantially influence which foods are produced in a given community. We focus on three potentially relevant measures: average temperature, average rainfall, and cluster altitude, where temperature and rainfall are measured as 30-year annual averages (1980–2010). We expect that more rainfall increases the array of crops that can be grown, as well as fodder for livestock, while agronomic research shows that high temperatures (e.g. above 29 degrees Celsius) reduce the yields of many crops, and will therefore prevent farmers from even attempting to grow heat-sensitive crops (Schlenker and Lobell, 2010). Temperature patterns and altitude also influence livestock diseases such as tsetse fly which restricts cattle ownership and therefore dairy and meat production.

### 2.2 Statistical analysis

Our analysis begins with descriptive evidence on consumption patterns in the full sample and the five major developing regions, before turning to non-parametric local polynomial smoothing regressions to examine the relationships between dietary diversity and various explanatory variables of interest. As there are many non-linear relationships, particularly for the community-level GIS indicators described above, we measure continuous indicators with terciles to flexibly and parsimoniously capture these non-linearities. We then use ordinary least squares regression models and linear probability models to estimate a regression model with DDS, MDD or consumption of a nutrient-rich food group as a function of DHS child and household characteristics (H), community characteristics (C), child age in month (Z), and country-year (survey) fixed effects ($\mu_{k}),$ where *i*, *j*, and *k* respectively denote child, cluster and country-year identifiers and ε is an error term:

(1) $$D_{i,j,k}=\beta_{H}H_{i,j,k}+\beta_{C}C_{j,k}+\delta Z_{i,j,k}+\mu_{k}+\varepsilon_{i,j,k}$$

The key parameters of interest in Eq. (1) are the coefficients captured by the vectors $\beta_{H}$ and $\beta_{C}$. Under the strong assumption that individual consumption is separable from home production, the household factors in Eq. (1) could be interpreted as shifting individuals' demand for each kind of infant food, while community factors capture shifts in market-level supply, demand and trade. In practice separability may not hold, so we interpret these associations as stylized facts that suggests avenues for further research. Since we control for country-year fixed effects, coefficients reflect only cross-sectional variation within each survey.

## 3 Main results

### 3.1 Descriptive results

Descriptive statistics for the key variables used in our regressions are reported in Table 2. Child dietary diversity score is low in this sample. Children consumed 2.81 out of 7 food groups, on average, in the past 24 h, and just 35% of children achieved MDD. Likewise, consumption of nutrient-rich food groups is low. For example, only 39% of children in this sample consumed dairy in the past 24 h. This is consistent with the generally low levels of development in the sample. Table 2 reports the wealth index scaled from 0 to 1 to facilitate interpretation, where 1 constitutes ownership of all assets. The average score is just 0.35, suggesting that most households only own a few of the assets that enter in to the wealth index. Moreover, only 39 percent of mothers and 47 percent of fathers have more than a primary level education, while only 27 percent of children have had access to the full measured spectrum of antenatal, neonatal and postnatal health care. We also report the raw measures of the continuous GIS variables, although the regression analysis below uses terciles. There is tremendous geographical variation in this sample of clusters, with a wide variety of agroecologies covered, as well as very different levels of infrastructure development.

**Table 2:** Descriptive statistics for the full sample of 76,641 children in 42 countries.

Variable	Mean	SD	Min	Max
**Child/parent indicators from DHS data**				
Wealth index, scaled 0–1	0.35	0.30	0.00	1.00
No maternal education	0.34	0.47	0.00	1.00
Maternal primary education (1–6 years)	0.27	0.45	0.00	1.00
Maternal secondary education (7–9 years)	0.15	0.36	0.00	1.00
Maternal tertiary education (10-plus years)	0.24	0.43	0.00	1.00
No paternal education	0.28	0.45	0.00	1.00
Paternal primary education (1–6 years)	0.26	0.44	0.00	1.00
Paternal secondary education (7–9 years)	0.15	0.35	0.00	1.00
Paternal tertiary education (10-plus years)	0.32	0.47	0.00	1.00
Healthcare access^a^	0.27	0.44	0.00	1.00
Child was breastfed immediately	0.66	0.47	0.00	1.00
Women can decide on own healthcare	0.62	0.49	0.00	1.00
Child is male	0.51	0.50	0.00	1.00
**Geographic characteristics of household locations**^b^				
Remote location (travel time to 20,000 person city > 1 hr)	0.50	0.50	0.00	1.00
Night lights intensity (index scale)	13.28	21.04	0.00	63.00
Population density (people per sq km)	735.18	2183.09	0.00	36904.40
Distance to coastline (km)	21.77	22.95	0.00	257.04
Distance to major inland water body (km)	360.53	367.94	0.00	1753.50
Mean rainfall per year, 1981–2010 (mm)	996.01	628.36	0.00	6127.83
Mean temperature, 1981–2010 (Celsius)	23.09	5.34	−4.66	30.20
Altitude (meters)	644.34	800.63	−377.00	4899.00

Note: DHS data are from Phase 5 & 6 surveys for 42 countries listed in supplemental Appendix Table A1; Geographic characteristics of household locations are computed from data sources described in the text, based on coordinates of DHS enumeration areas that are reported with systematic random error for de-identification.

a Healthcare access is equal to one if a child had prenatal care, was born in a medical facility and had the full set of recommended vaccinations.

b Geographical characteristics are drawn from IFPRI (2018).

Table 3 reports dietary patterns by region, while Appendix Figs. A1 and A2 reports mean DDS and MDD by country. Dietary diversity is lowest in sub-Saharan Africa (SSA), where just 22.1% of children achieve MDD. Consumption of most nutrient-rich foods is also low, although we note that fish consumption is exceptionally high by international standards, with one-quarter of African children consuming fish in the past 24 h, on par with dairy. Among vegetal foods, DGL vegetable consumption is relatively high in Africa. Patterns in the Asian sample are quite similar, with DGL vegetable consumption also high, and dairy and fish consumed by around one-quarter of Asian children. The other three regions have more diet diversity. In the Eastern Europe and Central Asia (ECA) region almost half of children achieve MDD and two-thirds consumed dairy and almost half consumed other fruits/vegetables, though very few consumed DGL or vitamin-A rich fruits/vegetables. Egg and meat consumption is also much higher in this region than in SSA or Asia. Patterns in Latin America and the Caribbean (LAC) and the Middle East and North Africa (MNA) are broadly similar to ECA, although DDS and MDD are higher, and there are one or two important differences for individual foods. In the small MNA sample, dairy consumption is very high (83.4%) and in LAC vitamin A-rich fruit/veg consumption is relatively high (39.9%). Overall, though, consumption of most nutrient-rich foods is much more prevalent in these three more developed regions than in SSA or Asia, with fish and DGL vegetables the main exceptions.

**Table 3:** Dietary diversity and intake of 8 food groups for infants 6–23 months of age, by region.

Regions^a^	DDS	MDD	DGL veg	Vit.A-rich fruit & veg^b^	Other fruit or veg	Legumes & nuts	Dairy	Eggs	Meat, organs	Fish^b^
SSA	2.3	22.1%	32.2%	14.9%	17.5%	24.5%	24.4%	11.7%	17.2%	24.6%
(N = 42,794)										
Asia	2.6	28.2%	42.0%	13.6%	21.4%	18.2%	28.7%	22.9%	21.4%	22.6%
(N = 7,968)										
ECA	3.1	45.2%	13.0%	18.9%	46.6%	15.4%	65.2%	37.0%	40.1%	3.7%
(N = 2,865)										
LAC	4.0	63.9%	17.6%	39.9%	47.5%	40.4%	64.0%	41.7%	55.8%	11.4%
(N = 17,658)										
MENA	3.6	56.3%	18.0%	10.5%	46.2%	28.4%	83.4%	46.5%	38.7%	14.4%
(N = 5,356)										
Total	2.8	35.2%	28.3%	27.7%	27.6%	27.3%	39.2%	22.8%	28.5%	20.4%
(N = 76,641)										

Note: Data shown use DHS household survey weights. Regional means other than Africa should be interpreted with caution due to small sample sizes, including just 4 countries for Asia, 3 for Eastern Europe and Central Asia, 7 for Latin America and the Caribbean, and just 2 for the Middle East and North Africa.

a Regional abbreviations are: SSA = sub-Saharan Africa; Asia refers to South Asia and South-East Asia; ECA = Eastern Europe and Central Asia; LAC = Latin America and the Caribbean; MENA = Middle East & North Africa;

b Latin America & Caribbean has only 12,963 observations for fish consumption due to missing data for Peru, while sub-Saharan Africa has only 40,985 observations for vitamin A-rich fruits due to lack of data for Tanzania. Standard deviation is in parenthesis.

### 3.2 Graphical results

**Fig. 1:**
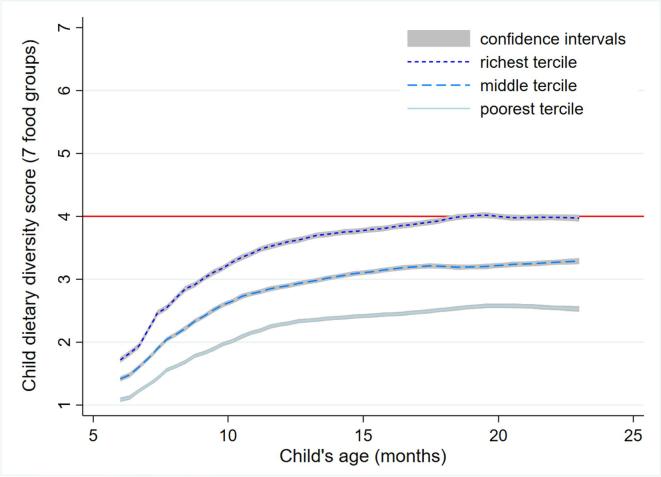
**Mean child dietary diversity score at each age in months, by wealth tercile.** Note: Data shown are local polynomial smoothing estimates with 95% confidence intervals (CI) for 76,641 infants aged 6–23 months with dietary intake data recorded in the Phase 5 & 6 surveys for 42 countries listed in supplemental Appendix Table A1, by tercile of household wealth computed as described in the text. The red line denotes the cut-off line for minimum dietary diversity (MDD). (For interpretation of the references to colour in this figure legend, the reader is referred to the web version of this article.)

Fig. 1 uses a non-parametric regression to demonstrate patterns of dietary diversity by child age, stratified by the lowest, richest and middle wealth terciles. Across all three terciles dietary diversity increases with age. At 6 months the diversity differences across wealth terciles are minimal as children are typically introduced to more palatable foods from just one or two groups (e.g. cereals, dairy). By 8 months, however, diversity differences across wealth terciles become stark and then widen and persist thereafter. It is also notable that even for the richest wealth tercile MDD is only achieved by around 18 months of age, on average.

In Appendix Fig. A3 we find a mostly linear relationship between dietary diversity and the raw wealth index score, consistent with Bennett’s observation that consumers diversify away from starchy staples as their incomes increase. There is some suggestion that the marginal effect of wealth may eventually decline, but in this relatively poor sample, the diminishing effects are modest. Nevertheless our regression estimates specify wealth terciles to allow for non-linear effects. In Appendix Fig. A4 we also observe strong but quite non-linear associations between dietary diversity scores and parental education, with a discrete break between having no education and any education, but also evidence of increasing returns to education with secondary school yielding much greater benefits than primary school (7 years or greater). We also observe a somewhat steeper gradient for maternal education. Both facts are consistent with Alderman and Headey (2018) findings about the associations between household variables and child stunting.

**Fig. 2:**
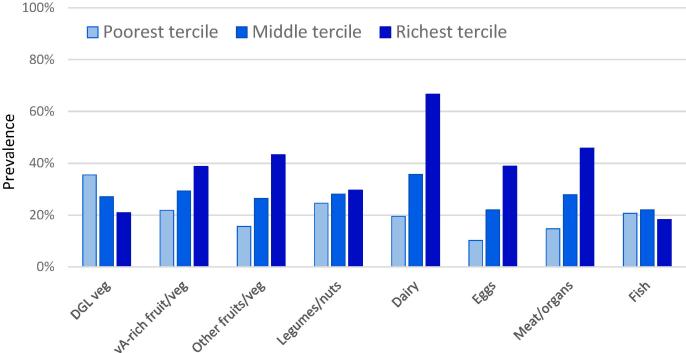
**Intake of 8 nutrient-rich food groups for children 6**–**23 months, by wealth tercile.** Note: Data shown are unweighted mean consumption prevalence of any food from each group in the past 24 h by terciles of the household wealth index described in Section 2, for children 6–23 months in 42 countries.

Fig. 2 shows the relationship between household wealth and the consumption of the eight nutrient-rich food groups described above, where wealth is split by terciles. For most nutrient-rich foods, consumption increases markedly with wealth, most strikingly for dairy, eggs, meat/organs and other fruits/veg. However, DGL vegetable intake declines as wealth increases, suggesting it is what economists refer to as an “inferior good” (as opposed to normal or luxury goods whose consumption rises with income). Fish consumption also declines slightly from the middle to richest tercile, and legume/nut consumption increases very slightly with wealth.

In Appendix Figs. A5 and A6 we report locally weighted regressions of the associations between dietary diversity and the community-level GIS indicators. These indicators have strikingly non-linear relationships. For example, being within one hour from a city/town of more than 20,000 people (20 K hereafter) is beneficial for dietary diversification, but that these benefits decline rapidly as the travel time extends beyond one hour. Rainfall is positively associated with dietary diversity until approximately 1300 mm per year, and thereafter flattens out. Average temperature is negatively associated with dietary diversity, although the relationship is non-linear. Night lights intensity shares a positive association with DDS, but the gradient eventually flattens. Distance to the coastline and to major inland water bodies shows no clear patterns, but population density is positively associated with diversity. These non-linearities prompt us to create a dummy if the cluster is more than one hour from a 20 K city/town to address the marked threshold at this cut-off, but to split the other indicators into terciles to capture these non-linearities.

### 3.3 Parametric multivariate regression results

Table 4 presents linear regression analysis of the determinants of DDS for children 6–23 months of age, as well as 12–23 months of age. This more restrictive sample is used because Fig. 2 demonstrated that the dietary benefits of wealth are minimal for infants 6–11 months who have only recently been introduced to solid foods. For the most part the regressions indicate that DDS results are broadly robust across these two age ranges, although in many cases the coefficients in the 12–23 month sample are larger in magnitude. We also note that we omit to report standard errors for the sake of brevity, although full results are provided in the Appendix (see Supplement C).

**Table 4:** Determinants of dietary diversity scores (DDS), by age.

	(1)	(2)
	6–23 months	12–23 months
Child/household indicators from the DHS		
Household wealth, middle vs low	0.146***	0.171***
Household wealth, high vs low	0.365***	0.424***
Maternal 1–6 yrs education vs none	0.127***	0.160***
Maternal 7–9 yrs education vs none	0.284***	0.342***
Maternal 10+ yrs education vs none	0.448***	0.518***
Paternal 1–6 yrs education vs none	0.097***	0.113***
Paternal 7–9 yrs education vs none	0.141***	0.151***
Paternal 10+ yrs education vs none	0.183***	0.207***
Health access vs none	0.218***	0.179***
Child breastfed immediately vs not	0.034***	0.051***
Mother decides own healthcare vs not	0.006	0.000
Child is male vs female	−0.002	−0.004
Geographic characteristics of household locations		
Remote location vs not remote	−0.031	−0.03
Night lights intensity, middle vs low	0.055**	0.056*
Night lights intensity, high vs low	0.143***	0.180***
Population density, middle vs low	0.045**	0.045**
Population density, high vs low	0.048*	0.054*
Distance to coastline, middle vs low	0.030	0.087***
Distance to coastline, high vs low	−0.013	0.020
Distance to water body, middle vs low	0.030*	0.027
Distance to water body, high vs low	0.028	0.014
Mean rainfall, middle vs low	0.122***	0.142***
Mean rainfall, high vs low	0.120***	0.111***
Mean temperature, middle vs low	−0.108***	−0.120***
Mean temperature, high vs low	−0.153***	−0.137***
Altitude by cluster, middle vs low	0.031	0.023
Altitude by cluster, high vs low	0.011	0.014
Age-in-month dummies included?	Yes	Yes
Country-year fixed effects included?	Yes	Yes
R-squared	0.356	0.311
N	76,641	49,123

Note: Significance levels shown are estimated using cluster-robust standard errors and are denoted: ***p < 0.01, **p < 0.05, *p < 0.1. Controls included but not reported are child age dummies, country and survey fixed effects.

Turning to the results, we find clear evidence for Bennett’s law applying to child diet diversity: the number of nutritionally-defined food groups fed to children for all ages rises linearly with each tercile. In the 12–23 month range, for example, the middle tercile consumes 0.15 food groups more than the poorest tercile, while the highest tercile consumed 0.42 food groups more relative to children from the poorest tercile. We also note that Appendix Table A2 reports the marginal effects in the MDD regression model, suggesting that children from the richest tercile are 12.3 points more likely to achieve MDD.

While these marginal wealth effects are reasonably large, the regressions also indicate that various knowledge proxies also play an equally important role in explaining variation in the dietary diversity of infants. Women with 10 or more years of education are likely to feed their children an extra 0.5 food groups, and 13.3 points more likely to achieve MDD (Appendix Table A2). Interestingly, the corresponding marginal effects for paternal education – though still highly significant – are less than half the size of the maternal education effects. Further indirect evidence of a role for nutritional knowledge is reflected in the coefficient on health access, which is associated with an extra 0.2 food groups and a 5–7 point increase in the likelihood of achieving MDD (Appendix Table A2). However, we find no statistically significant coefficients associated with a mother’s ability to make her own healthcare decisions.

In addition to household level factors, indicators of geographical and infrastructural characteristics share a number of significant associations with child dietary diversity scores. Interestingly, more remote locations share no sizeable and significant association with dietary diversity once other characteristics are controlled for. In contrast, the night lights intensity index – which is associated with economic development, electrification and urbanization - has a strong association with DDS. Children in high intensity communities are predicted to consume an extra 0.14–0.18 food groups, and 3–4 points more likely to achieve MDD (Appendix Table A2). Population density is also significantly associated with DDS, though the marginal effect sizes are more modest. Coastal access has modest associations with these dietary metrics, as does access to inland water bodies. In contrast, there are clear signs of a significant dietary penalty associated with living in low rainfall communities, since the lowest rainfall tercile is likely to consume 0.11–0.14 fewer food groups than middle and high rainfall communities. There is a penalty of similar magnitude for being in cooler locations, but no significant association between altitude and dietary diversity measures.

Table 5 reports results with consumption of various nutrient-rich vegetal foods as the dependent variable. Consistent with Fig. 2, DGL vegetable consumption does not rise with wealth, nor does legume/nut consumption. However, children from the richest wealth tercile are 5–6 times more likely to consume vitamin-A rich fruits/vegetables or other fruit/vegetables. Interestingly, however, more educated mothers and fathers are more likely to feed their children all of these foods, though there are no clear signs of increasing returns. Health service access is also associated with modest increases in all foods except DGL vegetables. Amongst the community-level GIS variables, consumption of fruits and vegetables tends to be higher in communities further away from the coast and from water bodies. For all crops there is a clear penalty to residing in the coolest temperature tercile, but also in the lowest rainfall tercile.

**Table 5:** Determinants of intake by vegetal food group, for children 6–23 months.

	(1)	(2)	(3)	(4)
	DGL Veg	vA-rich fruit, veg	Other fruit, veg	Legumes, nuts
Child/household indicators from the DHS				
Household wealth, middle vs low	−0.016***	0.013***	0.021***	0.016***
Household wealth, high vs low	−0.009	0.056***	0.057***	0.010
Maternal 1–6 yrs education vs none	−0.007	0.006	0.016***	0.019***
Maternal 7–9 yrs education vs none	0.010	0.029***	0.047***	0.037***
Maternal 10 + yrs education vs none	0.029***	0.057***	0.076***	0.034***
Paternal 1–6 yrs education vs none	0.006	0.007	0.005	0.029***
Paternal 7–9 yrs education vs none	0.008	0.017***	0.017***	0.014**
Paternal 10+ yrs education vs none	0.014**	0.024***	0.024***	0.015***
Health access vs none	−0.002	0.024***	0.048***	0.012***
Child breastfed immediately vs not	0.003	0.007**	0.008**	0.009**
Mother decides own healthcare vs not	0.009**	−0.005	−0.005	0.003
Child is male vs female	0.000	0.000	0.002	−0.001
Geographic characteristics of household locations				
Remote location vs not remote	−0.006	−0.012**	−0.011**	0.002
Night lights intensity, middle vs low	−0.005	0.017**	0.001	−0.017***
Night lights intensity, high vs low	0.000	0.009	0.016**	−0.005
Population density, middle vs low	0.005	0.008	0.024***	0.004
Population density, high vs low	0.01	0.023***	0.026***	0.009
Distance to coastline, middle vs low	0.004	0.006	−0.012**	0.010*
Distance to coastline, high vs low	0.075***	−0.016*	−0.022***	0.007
Distance to water body, middle vs low	0.023***	0.014***	0.011**	0.007
Distance to water body, high vs low	0.029***	0.012**	−0.004	0.020***
Mean rainfall, middle vs low	0.008	0.007	0.033***	0.031***
Mean rainfall, high vs low	−0.015*	−0.012	0.033***	0.030***
Mean temperature, middle vs low	−0.028***	−0.043***	−0.009	−0.020***
Mean temperature, high vs low	−0.058***	−0.057***	−0.030***	−0.044***
Altitude by cluster, middle vs low	0.014**	0.007	0.006	0.005
Altitude by cluster, high vs low	0.022***	−0.013	0.008	0.033***
Age-in-month dummies included?	Yes	Yes	Yes	Yes
Country-year fixed effects included?	Yes	Yes	Yes	Yes
R-squared	0.169	0.144	0.183	0.154
N	76,641	76,641	76,641	76,641

Note: Significance levels shown are estimated using cluster-robust standard errors and are denoted: ***p < 0.01, **p < 0.05, *p < 0.1. Controls included but not reported are child age dummies, country and survey fixed effects.

Table 6 reports analogous results for consumption of animal-sourced foods. Also consistent with Fig. 2, dairy consumption rises starkly with wealth. Children from the highest wealth tercile are 13 points more likely to consume dairy, compared to an 8.6 point increase in meat/organ consumption and a 5.7 point increase in egg consumption. However, fish consumption does not rise with wealth, which again suggests that fish – though often highly nutritious – may be regarded as an inferior good. Indeed, the overall pattern of wealth effects suggest that as households become richer they substitute out of fish and into other ASFs. Parental education is again significantly associated with consumption of nutrient-rich animal-sourced foods, although the effects are stronger for maternal education and highest for dairy. Health access is also associated with consumption of all ASFs, except fish.

**Table 6:** Determinants of intake by animal-sourced food group, for children 6–23 months.

	(1)	(2)	(3)	(4)
	Dairy	Eggs	Meat, organs	Fish^b^
Child/household indicators from the DHS				
Household wealth, middle vs low	0.041***	0.028***	0.031***	0.011***
Household wealth, high vs low	0.131***	0.057***	0.086***	0.000
Maternal 1–6 yrs education vs none	0.014***	0.016***	0.009**	0.041***
Maternal 7–9 yrs education vs none	0.064***	0.033***	0.036***	0.037***
Maternal 10 + yrs education vs none	0.114***	0.055***	0.077***	0.034***
Paternal 1–6 yrs education vs none	−0.005	0.015***	0.005	0.032***
Paternal 7–9 yrs education vs none	0.019***	0.023***	0.021***	0.028***
Paternal 10+ yrs education vs none	0.028***	0.032***	0.020***	0.043***
Health access vs none	0.053***	0.052***	0.050***	0.006
Child breastfed immediately vs not	−0.006	0.017***	0.012***	0.009***
Mother decides own healthcare vs not	−0.016***	0.001	−0.003	0.006*
Child is male vs female	0.007**	−0.004	0.000	0.000
Geographic characteristics of household locations				
Remote location vs not remote	0.012**	−0.005	−0.007	−0.009*
Night lights intensity, middle vs low	0.027***	0.007	0.029***	0.007
Night lights intensity, high vs low	0.069***	0.029***	0.050***	−0.001
Population density, middle vs low	−0.007	−0.004	0.015***	−0.012**
Population density, high vs low	0.000	−0.005	0.006	−0.026***
Distance to coastline, middle vs low	0.014**	0.011**	0.019***	−0.013**
Distance to coastline, high vs low	0.023**	−0.020***	0.017**	−0.037***
Distance to water body, middle vs low	−0.002	0.011***	0.008*	−0.042***
Distance to water body, high vs low	−0.004	0.010**	0.015***	−0.052***
Mean rainfall, middle vs low	−0.044***	0.021***	0.022***	0.045***
Mean rainfall, high vs low	−0.064***	0.044***	0.022***	0.099***
Mean temperature, middle vs low	−0.009	−0.017***	−0.001	0.025***
Mean temperature, high vs low	−0.008	−0.020**	0.024***	0.051***
Altitude by cluster, middle vs low	−0.009	0.021***	0.013**	−0.045***
Altitude by cluster, high vs low	0.001	0.028***	−0.032***	−0.057***
Age-in-month dummies included?	Yes	Yes	Yes	Yes
Country-year fixed effects included?	Yes	Yes	Yes	Yes
R-squared	0.276	0.187	0.232	0.163
N	76,641	76,641	76,641	70,137

Note: Significance levels shown are estimated using cluster-robust standard errors and are denoted: ***p < 0.01, **p < 0.05, *p < 0.1. Controls included but not reported are child age dummies, country and survey fixed effects.

Turning to community factors, night lights intensity is again positively associated with greater ASF consumption, except fish. Distance to the coastline and to major water bodies have weak associations with ASF consumption in general, although both indicators are strong predictors of fish consumption. For example, children close to major water bodies are 4–5 points more likely to consume fish. Amongst climate variables, drier conditions are associated with more dairy consumption, but less egg, meat and fish consumption, while temperature effects vary in sign and magnitude. Altitude generally has modest associations, although children living in low altitude localities are significantly more likely to consume fish and less likely to consume eggs. Overall there is fairly strong evidence that agroecological conditions shape which ASFs children are fed in developing countries.

## 4 Extensions

### 4.1 Sensitivity to the exclusion of household or community level variables

Appendix Table A3 gauges the sensitivity of the 12–23 month DDS results to the exclusion of household or community level variables. We do this for two reasons. First, previous studies of diets in the DHS, such as Senarath et al., 2012, Headey et al., 2018b, have only used household level indicators from the DHS itself, so it is important to assess the sensitivity of these results to the inclusion of GIS indicators. Second, it is probable that many of the community level factors influence diets through household-level factors. For example, one would expect agroecological conditions to be important predictors of household wealth. It is therefore interesting to assess how these factors influence dietary diversity when household level factors are potential mediators.

Appendix Table A3 first reports the full model reported in regression (2) of Table 2 as a benchmark, while regressions (2) and (3) in Appendix Table A3 exclude community- and household-level factors, respectively. In regression (2) we observe that the exclusion of community-level factors generally has modest impacts on the household-level coefficients, except for the wealth coefficients, which increase quite substantially with the exclusion of GIS variables. The high vs low wealth effect increases from 0.424 to 0.548, for example.

In regression (3) of Appendix Table A3 we see that excluding household level factors results in quite large changes in the marginal effects of many GIS indicators. For example, children in remote locations consume 0.14 fewer food groups, and the coefficients on night lights intensity terciles roughly triple in magnitude. Population density coefficients also increase, and children in clusters far away from the coast now consume 0.16 fewer food groups. There are also larger benefits to higher rainfall and greater costs to higher temperatures.

One important explanation of this pattern of results is that many of these GIS-level indicators are reasonably strong predictors of household wealth, parental education and access to health services. To examine this we estimated regressions with household wealth as the dependent variable and the various GIS indicators as explanatory variables. Coefficient plots with 95% confidence intervals are reported in Appendix Fig. A7. Most striking, but not unexpected, is the strong association between night lights intensity and household wealth. Indeed, a regression of wealth against night lights intensity, without any other controls, explains around two-thirds of the variation in household wealth. Yet many other GIS variables explain household wealth. Populations that are more remote from cities and the coastline are poorer, as are populations in warmer and drier places.

Next, Table 7 examines the ability of the different variables in regressions (1), (2) of Appendix Table A3 to predict variation in child DDS. To do this we conduct a simple regression-decomposition between groups, as in previous analyses of decompositions of nutrition change over time (Headey et al., 2015) or space (Cavatorta et al., 2015). Here we decompose the DDS differences between nine high-diversity countries with MDD prevalence of 40% or higher (the top nine countries in Appendix Fig. A2) and the remaining 33 low-diversity countries with MDD prevalence of 33% or less. In effect, the regression decomposition asks what the change in DDS would be if the low diversity countries had the mean levels of household level variables (***H***) or community-level variables (***C***) that the high diversity country have. Hence the predicted difference in DDS between high and low diversity countries due to any specific variable is just the product of the relevant regression coefficient and the difference in means across the two samples:

(2) $${\Delta D}_{i,j,k}=\beta_{H}{\Delta H}_{i,j,k}+\beta_{C}\Delta C_{j,k}$$

**Table 7:** Using regression decomposition at variable means to explain differences in dietary diversity scores.

	(1)	(2)
	Full model	Child & household variables only
	Predicted difference in DDS	
Wealth	0.16	0.23
Parental education	0.20	0.25
Health access	0.05	0.04
Night lights	0.06	NA
Climate variables	0.03	NA
	Predictive power	
Actual difference in DDS between high and low diversity countries	1.57	1.57
Total explained difference between high and low diversity countries (% of actual)	0.49 (31.2%)	0.53 (33.5%)

Notes: High diversity countries are those where MDD prevalence is greater than 40%. This includes Peru, Dominican Republic, Albania, Bolivia, Honduras, Colombia, Jordan, Guyana and Egypt (see Fig. A2 for MDD prevalence by country). The table reports a decomposition at means. In column (1) the predicted change in DDS is the product of coefficients reported in regression (1) of Appendix Table A3 and the difference in means between a sample of low diet diversity countries and sub-sample of high dietary diversity countries. Column (2) uses the coefficients from regression (2) of Appendix Table A3, which excludes community level variables.

The results in Table 7 allow us to gauge the predictive importance of individual variables but also the accuracy of the model as a whole. In column (1) we see that inter-group differences in wealth account for a 0.16 difference in DDS, while parental education accounts for 0.20, with health access, night lights and climate variables explaining a further 0.14 difference collectively. This sums to a 0.40 difference in DDS, which is only around one-third of the actual difference in DDS across the sample. In column (2) we conduct a decomposition based on regression (2) in Appendix Table A3 where community-level factors were omitted. This increases the contribution of wealth differences to 0.23 and education differences to 0.25, with health access still making only a small contribution, and the model as a whole still accounting for one-third of the inter-group difference.

Two important conclusions therefore stem from Table 7. First, the model as a whole clearly does not fully explain why these nine countries have substantially more dietary diversity than the remaining country; and country-specific factors likely play an important role. Second, household wealth is not the paramount driver of dietary diversification that it is often assumed to be (at least, by economists); parental education is at least as important in a purely statistical sense.

### 4.2 Regional heterogeneity

Next, we exploit the substantive geographical coverage in our data to explore heterogeneity of the DDS results across regions in Table 8. Results across the two least developed regions, SSA and Asia, are relatively similar, although there are somewhat larger marginal effects for paternal education and health access in Asia. Wealth and maternal education effects in LAC are somewhat larger in magnitude, but the coefficients on paternal education in this region are not statistically different from zero. In MNA and ECA, wealth effects are insignificantly different from zero, and there are many fewer significant coefficients in general, perhaps reflecting higher standards of living in these regions.

**Table 8:** Heterogeneity in determinants of dietary diversity scores, for children 6–23 months by region.

**Panel A: Child/household indicators**					
	(1)	(2)	(3)	(4)	(5)
	Sub-Saharan Africa	Asia	Latin America & Caribbean	Middle East & North Africa	East Europe & Central Asia
Child/household indicators from the DHS					
Middle wealth vs low	0.032***	0.052***	0.077***	−0.075	0.065
Upper wealth vs low	0.082***	0.092***	0.147***	0.052	0.06
Maternal primary education	0.011**	0.043***	0.090***	0.057**	0.066
Maternal secondary education	0.052***	0.065***	0.151***	0.072**	0.027
Maternal tertiary education	0.091***	0.101***	0.176***	0.132***	0.042
Paternal primary education	0.013**	0.033**	0.018	0.019	0.008
Paternal secondary education	0.021***	0.059***	0.022	0.006	−0.019
Paternal tertiary education	0.043***	0.100***	0.02	0.044*	−0.004
Health access	0.061***	0.112***	0.057***	0.026	0.059***
Child was breastfed immediately	0.016***	0.011	0.007	0.008	0.023
Mother can decide on own healthcare	−0.012**	0.026**	0.01	−0.026	−0.015
Child is male	−0.003	0.008	0.007	−0.002	−0.017
N	42,794	7,968	17,658	5,356	2,865

**Panel B: Geographic characteristics**					
	Sub-Saharan Africa	Asia	Latin America & Caribbean	Middle East & North Africa	East Europe & Central Asia
Geographic characteristics of household locations					
Remote location vs not remote	−0.006	−0.031**	−0.016	0.013	0.003
Night lights intensity, middle vs low	0.009	−0.004	0.005	0.088	0.024
Night lights intensity, high vs low	0.048***	0.014	−0.017	0.176**	0.042
Population density, middle vs low	0.002	0.026	0.039***	0.021	−0.009
Population density, high vs low	0.011	0.023	0.041***	0.005	−0.017
Distance to coastline, middle vs low	0.033***	−0.014	0.034***	−0.044***	
Distance to coastline, high vs low	0.034***	−0.035	−0.043**		
Distance to water body, middle vs low	0.009	0.026*	−0.001	0.014	−0.036
Distance to water body, high vs low	0.005	0.003	−0.005	0.073***	−0.048
Mean rainfall, middle vs low	0.022***	0.087	0.018		−0.069
Mean rainfall, high vs low	0.055***	0.041	−0.006		
Mean temperature, middle vs low	−0.038***	−0.059**	−0.009	0.023	
Mean temperature, high vs low	−0.022*	−0.039	−0.051***	−0.012	
Altitude by cluster, middle vs low	−0.005	−0.009	0.005	0.046	−0.074
Altitude by cluster, high vs low	0.025*	−0.050**	−0.039***	0.067*	−0.112**
Age-in-month dummies included?	Yes	Yes	Yes	Yes	Yes
Country-year fixed effects?	Yes	Yes	Yes	Yes	Yes
R-squared	0.165	0.131	0.204	0.114	0.099
N	42,794	7,968	17,658	5,356	2,865

Notes: Cluster-robust standard errors were used to estimate significance levels denoted: ***p < 0.01, **p < 0.05, *p < 0.1. Controls included but not reported are child age dummies, country and survey fixed effects.

For the GIS indicators there are limits in the number of clusters in most regions that likely restrict the ability of these regressions to accurately identify community-level effects, so we focus the discussion here on SSA, which has a large sample size and ample geographic variation in these indicators. In SSA we again observe positive associations between night lights intensity and DDS, but also that areas further from the coast have somewhat higher DDS. Given the region’s vulnerability to climate change, the sensitivity of DDS to rainfall and temperature is particularly striking. We also note that Appendix Tables A4 and A5 report SSA-specific results for determinants of the eight nutrient-rich food groups. These regressions also confirm that low rainfall and high temperatures are associated with reduced intake of the various fruits and vegetables, legumes/nuts, eggs and meat/organs, though not fish or dairy products. We note that we also experimented with an alternative measure of climate, the length of the growing season, which is strongly correlated with rainfall (r = 0.79). This variable yielded similarly strong results, with lengthier growing periods generally associated with greater dietary diversity and increased consumption of vegetal foods, in particular (results not shown).

In Appendix Table A6 we compare the wealth effects estimated from food group regressions for Africa (SSA plus Egypt) to the median income elasticities reported in a recent meta-analysis of these parameters for Africa (Colen et al., 2018). The regression specifications used to derive our child-level results are very different to those used in household food consumption studies that populated the Colen et al. meta-analysis, meaning that the coefficients in question cannot be directly compared in magnitude. Nevertheless, the patterns of coefficient magnitudes are actually very similar. The correlation between middle vs low wealth effects and the Colen et al. median income elasticities is 0.67, while the corresponding correlation for high vs low wealth effects is 0.86. In both sets of results, income/wealth effects for vegetal foods are generally low (and mostly statistically insignificant from zero in our regressions), while income/wealth elasticities for ASFs are generally high. However, our results also point to the need to look at more disaggregated food groups. As we saw above, wealth effects on DGL vegetables are actually negative, while the wealth effect on fish consumption is about one-fifth of the high versus low wealth effect estimated for meat, and one third of the corresponding effect for eggs. This is a potentially important finding given that fish is the most commonly consumed ASF in SSA, and in some parts of Asia, and that fish are rich in protein and a range of micronutrients.

## 5 Conclusions

Economic analysis of food choice and diet quality is usually confined to purchases by households, rather than individual intake, especially regarding infants from 6 to 24 months when growth faltering most often occurs. We overcome past data constraints on infant feeding by combining data from 42 countries covered by the DHS with a rich array of GIS-based community level data that capture economic and infrastructural development as well as exogenous agricultural conditions. This extensive dataset allows us to document diverse child feeding patterns across regions and economic strata, to estimate household and community level determinants with precision and flexibility, and to explore heterogeneity in these associations across developing regions. While the DHS dietary data are limited insofar as they do not provide quantities, their high degree of standardization allow us to analysed consumption patterns for a critically important age group.

In this section we flag some of the most important findings from our results, and discuss their implications for new research and for nutrition-smart policies designed to improve child feeding.

### 5.1 Wealth, nutritional knowledge and dietary diversification

We find a highly significant and approximately linear association between child diet diversity and household wealth, providing strong support for an alternative version of Bennett’s law defined by food group diversity (rather than calorie shares). The marginal effect of household wealth is about the same size as the marginal effect associated with 10+ years of maternal education. Wealth and education account for similar fractions of the difference between low and high dietary diversity populations, which may partially account for the robust association between maternal education and stunting or other nutrition outcomes (Alderman and Headey, 2018). It is possible that maternal education represents unobserved wealth, and likely that it partly reflects her empowerment, but also probable that associations with dietary diversity are substantially driven by knowledge and preferences. Similarly, health access might partly reflect wealth insofar as health services are income-elastic but might also proxy for parents accessing health/nutrition information, as could breastfeeding in the first hour after birth. In summary, a view that household wealth is the only driver of diet quality clearly does not apply to these data; for infant feeding, maternal education and the food environment are also important correlates of how well a child's nutritional needs are likely to be met.

### 5.2 Community-level characteristics and child nutrition

We offer novel evidence that indicators of geographic, demographic and infrastructural characteristics – as well as night lights (which likely reflects broader local economic development) – have significant and reasonably large associations with child dietary diversity scores. Night lights intensity is strongly associated with household wealth, suggesting that it can serve as a general indicator of economic prosperity (Henderson et al., 2012) as well as an indicator for one specific kind of infrastructure. In terms of urbanization and other geographical factors, proximity to a city had only weak associations with dietary diversity but population density had moderately strong associations (similar to Headey et al., 2018b), while distances to the nearest coast and major inland water body were associated with increased consumption of some specific foods (especially fish), but not with overall dietary diversity.

Of significant policy relevance in the context of a rapidly warming and more volatile climate, we find strong relationships between climate indicators and dietary diversity, as infants in the lowest tercile of temperatures and highest tercile of rainfall have the most diverse diets. This is consistent with a wide range of previous research suggesting that more temperate conditions facilitate agricultural growth and broader development (e.g. Masters and McMillan, 2001), and that individual crop yields are highly sensitive to extreme heat during the growing season, specifically temperatures in excess of 29 degrees Celsius for many cereals such as maize, wheat, sorghum and millet (Schlenker and Lobell, 2010). A recent systematic review highlights the impacts of climate change on yields and nutritional quality of vegetables (Scheelbeek et al., 2018). Our evidence is consistent with a strong connection between temperature and consumption of plant-based non-staple foods: children in the hottest temperature tercile are 3–6 points less likely to consume a fruit, vegetable, or legume. This raises the possibility that future climate change will further reduce consumption of nutrient-rich vegetal products. For livestock, climate connections are much less clear, and it is possible that climate changes could precipitate substitution within crops and between crops and livestock.

### 5.3 Children's consumption of specific nutrient-rich foods

A subtle but important finding of our research is that while overall dietary diversity increases with wealth (and parental education), different kinds of nutrient-rich foods vary differently with household wealth and aspects of the child's environment. At one extreme, dairy consumption rises sharply with household wealth and night lights intensity. The wealth gradient for meat is also relatively steep, while the gradient for eggs is more modest, and fish consumption does not rise with household wealth and is quite prevalent even among the lowest wealth tercile. In terms of vegetal foods, vitamin A-rich fruits and vegetables and other fruit/vegetables have modest wealth gradients similar to egg consumption, with children from the wealthiest tercile about 5.7 times more likely to consume these products than children from the poorest tercile of household wealth. However, legume/nut consumption is largely invariant to wealth, and DGL vegetable consumption actually declines with wealth. We note that these results are broadly consistent with economic studies of household level demand for these foods (see Supplement B).

The fact that parental use of some healthy foods such as DGL vegetables declines with wealth, and that there is no income effect for fish, suggests a particularly important role for nutritional knowledge and preferences regarding these foods, and possibly a role for food preparation and time constraints as caregivers switch to other nutrient-dense foods notably eggs, meat or dairy. Since DGL vegetables and fish could provide valuable complements to other foods, parents might be encouraged to keep these healthy foods in child diets even as they add other food groups over time. Again, studying the evolution of infant diets in developing countries is an important area for future research.

### 5.4 Strengths and limitations

All results in this study are purely observational, intended to establish novel stylized facts about the timing and degree of diversification in children’s diets in low- and middle-income countries. The DHS data we use provide nationally-representative samples of unprecedented size and scope, but are necessarily limited to a dichotomous indicator for intake of major food groups over the previous day or night. This single 24-hour food frequency questionnaire is less precise than more time-intensive forms of dietary assessment, and does not allow analysis of seasonal or other variation in feeding practices. Furthermore, our analysis focuses on the food groups used for dietary diversity scores, omitting premixed cereals or other packaged products. The potential correlates of feeding practices that we examine are limited to a relatively simple wealth index, and no direct measures of nutritional knowledge or exposure to programs designed to improve that knowledge. Finally, our analysis aims to establish global patterns, with only suggestive evidence about regional heterogeneity. Future work could aim to overcome many of these limitations, building on the steps taken in this study to understand and improve the diets of young children which are particularly important for physical and cognitive development (Glewwe et al., 2001, Horton and Alderman, 2008, Hoddinott, 2009). No previous study of which we are aware has extensively examined both household and geographical predictors of infant food consumption at a global scale, although Galway et al. (2018), Headey et al. (2018a) and many others do address a variety of specific questions around children’s diets.

Overall, our findings have important implications for future research and policy design, especially for countries with the highest burdens of malnutrition. Our results suggest that while wealth accumulation is indeed an important driver of diversification, women’s education and the food environment are likely to play an additional independent role as well. Access to maternal and child health care may also play an important role in how children are fed, highlighting opportunities to improve the nutritional messaging of conventional health services (Menon et al., 2015). All of these findings involve complex bidirectional relationships, heterogeneity and other factors to be investigated in future studies of infant feeding practices. Future studies might also examine the impacts of expanding access to premixed cereals and other packaged foods that are not included in our measure of dietary diversification, but may be widely used for infant feeding even in rural areas. Finally, the associations we find between climate and infant feeding patterns warrant further investigation, including how climate change will alter availability and use of each food group needed to prevent child malnutrition.
