# Supplementary material for: First foods: Diet quality among infants aged 6–23 months in 42 countries
Source: Food Policy. 2019 Oct;88:101762. doi: 10.1016/j.foodpol.2019.101762 (PMC6894322; doi:10.1016/j.foodpol.2019.101762)
Supplement: Supplementary data 1 [file mmc1.docx]

**Supplemental material for S. Choudhury, D. Headey and W.A. Masters (2019),**

**“First foods: Diet quality among infants aged 6–23 months in 42 countries”**

This version revised 2 September 2019

Contact author: [william.masters@tufts.edu](mailto:william.masters@tufts.edu)

**Appendix A**

**Table A1. Countries, years and sample sizes of DHS surveys, phase 5 and 6**

| **Survey** | **N** |  | **Survey** | **N** |
| --- | --- | --- | --- | --- |
| **Sub-Saharan Africa (SSA)** |  |  | **Asia** |  |
| Benin 2011/2012 | 2,907 |  | Bangladesh 2011/2011 | 2,293 |
| Burkina Faso 2010/2010 | 1,843 |  | Cambodia 2010/2010 | 1,080 |
| Burundi 2010/2010 | 965 |  | Nepal 2006/2006 | 1,515 |
| Cameroon 2011/2011 | 1,423 |  | Nepal 2011/2011 | 664 |
| Comoros 2012/2012 | 541 |  | Timor-Leste 2009/2010 | 2,416 |
| Cote d'Ivoire 2011/2012 | 788 |  |  |  |
| Ethiopia 2011/2011 | 2,473 |  | **Eastern Europe and Central Asia (ECA)** | |
| Gabon 2012/2012 | 664 |  | Albania 2008/2009 | 370 |
| Ghana 2008/2008 | 695 |  | Kyrgyz Republic 2012/2012 | 1,203 |
| Guinea 2012/2012 | 919 |  | Tajikistan 2012/2012 | 1,292 |
| Lesotho 2009/2009 | 144 |  |  |  |
| Liberia 2013/2013 | 748 |  | **Latin America & Caribbean (LAC)** | |
| Madagascar 2008/2009 | 1,176 |  | Bolivia 2008/2008 | 2,065 |
| Malawi 2010/2010 | 1,415 |  | Colombia 2010/2010 | 3,952 |
| Mali 2012/2013 | 1,317 |  | Dominican Republic 2007/2007 | 1,881 |
| Mozambique 2011/2011 | 2,546 |  | Dominican Republic 2013/2013 | 596 |
| Namibia 2006/2007 | 597 |  | Guyana 2009/2009 | 282 |
| Namibia 2013/2013 | 200 |  | Haiti 2005/2006 | 671 |
| Nigeria 2008/2008 | 2,654 |  | Haiti 2012/2012 | 1,039 |
| Nigeria 2013/2013 | 7,563 |  | Honduras 2011/2012 | 2,477 |
| Rwanda 2010/2010 | 923 |  | Peru 2004/2008 | 1,990 |
| Senegal 2010/2011 | 1,083 |  | Peru 2009/2009 | 2,705 |
| Senegal 2012/2013 | 1,571 |  |  |  |
| Sierra Leone 2008/2008 | 505 |  | **Middle East & North Africa (MENA)** | |
| Sierra Leone 2013/2013 | 1,101 |  | Egypt 2008/2008 | 3,070 |
| Swaziland 2006/2007 | 359 |  | Jordan 2007/2007 | 568 |
| Tanzania 2010/2010 | 1,809 |  | Jordan 2012/2012 | 1,718 |
| Uganda 2006/2006 | 608 |  |  |  |
| Uganda 2011/2011 | 543 |  |  |  |
| Zambia 2007/2007 | 1,448 |  |  |  |
| Zimbabwe 2010/2011 | 1,266 |  | **Total** | **76,641** |

**Figure A1. Mean dietary diversity scores by country**

**Figure A2. Minimum dietary diversity prevalence by country**

**Figure A3. Mean child dietary diversity score by level of household wealth**


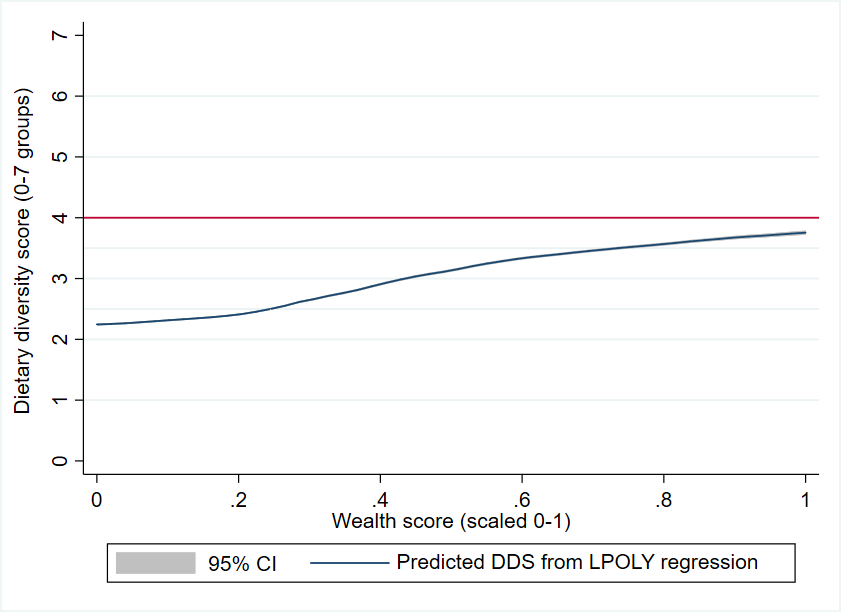


Note: Data shown are local polynomial smoothing estimates with 95% confidence intervals (CI) for 76,641 infants aged 6-23 months with dietary intake data recorded in the Phase 5 & 6 surveys for 42 countries listed in supplemental appendix Table A1, by household wealth computed as described in the text. The red line denotes the cut-off line for minimum dietary diversity (MDD).

**Figure A4. Nonparametric estimates of the relationship between child dietary diversity score and years of parental education**


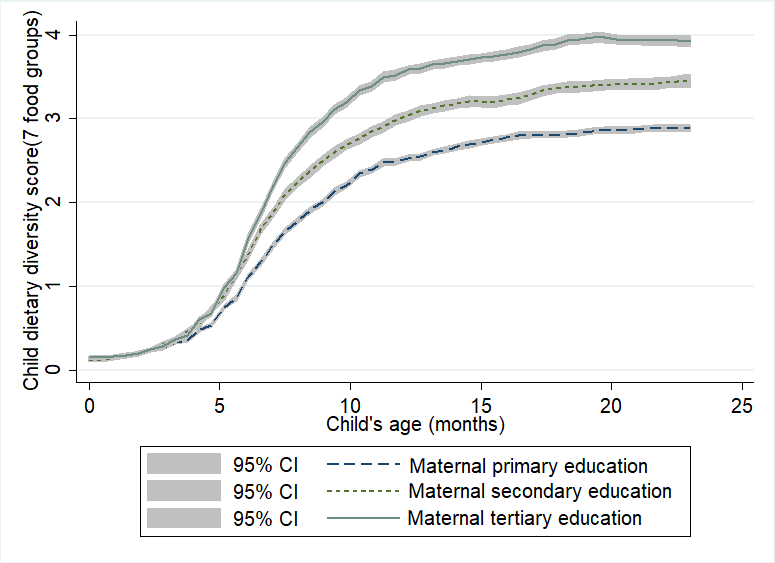


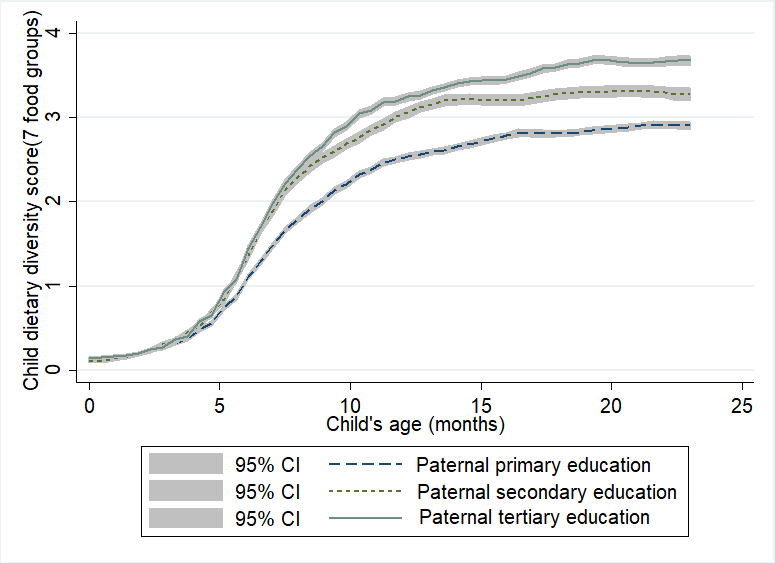


Source: Phase 5 & 6 DHS data for 42 countries. These are local polynomial smoothing estimates with 95% confidence intervals (CI)

**Figure A5. Dietary diversity scores and GIS-based agro-ecological indicators (LPOLY plots with 95% CIs)**

| (a) Average rainfall over 30 year period | (b) Average temperature over the 30 year period |
| --- | --- |


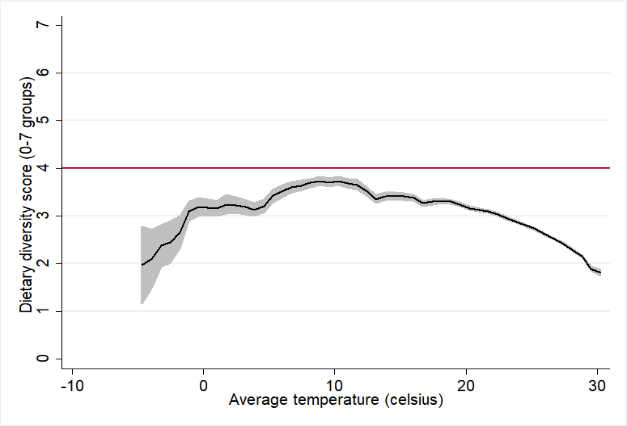

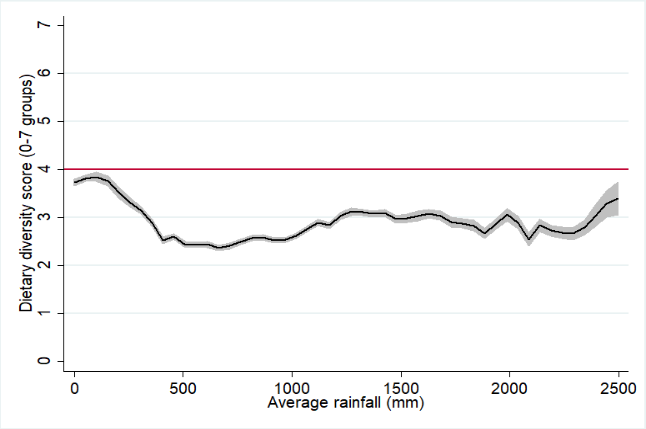


| (c) Altitude (meters)  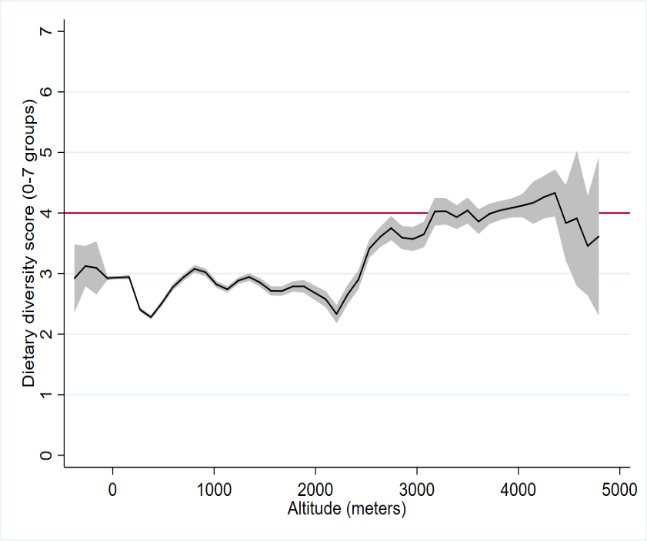 | (d) Distance to major inland water body (km)  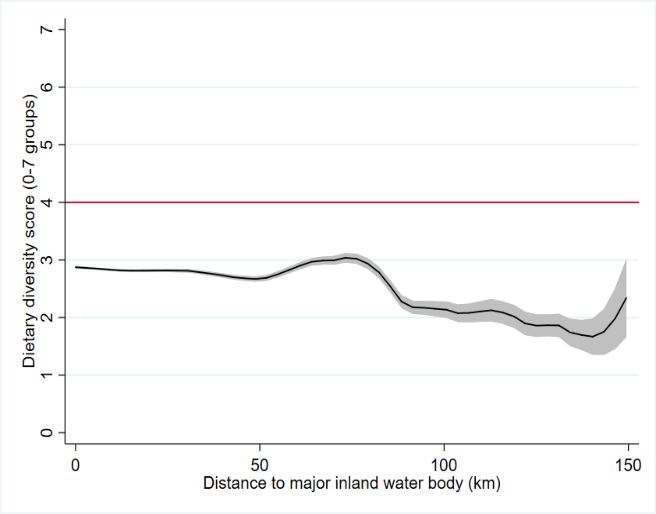 |
| --- | --- |

Source: Phase 5 & 6 DHS data for 42 countries. These are local polynomial smoothing estimates with 95% confidence intervals (CI)

**Figure A6. Dietary diversity scores and GIS-based infrastructural indicators**

| (a) Night lights intensity index | (b) Travel time to cities greater than 20k population |
| --- | --- |


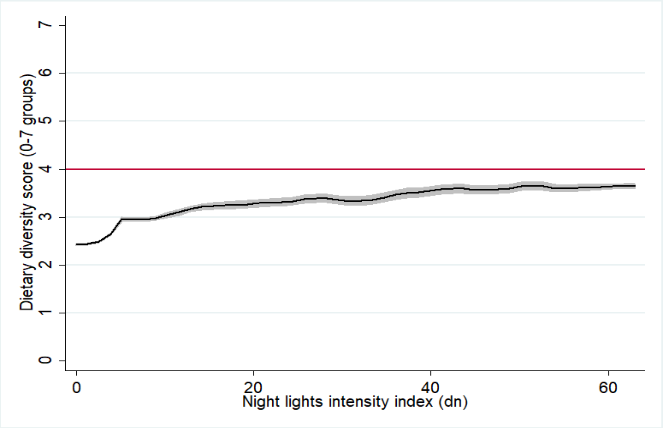

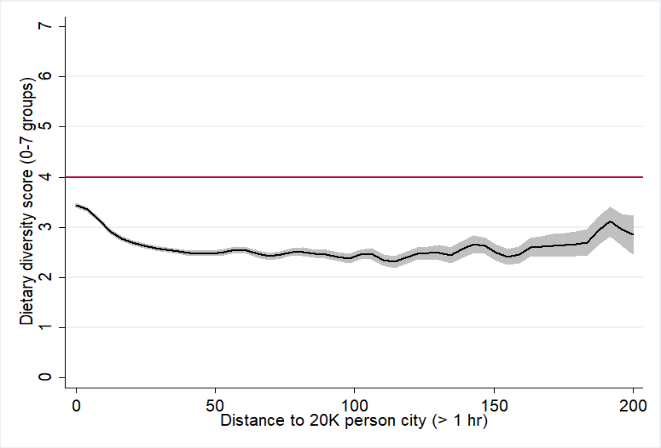


| (c) Distance to coastline | (d) Total population density |
| --- | --- |


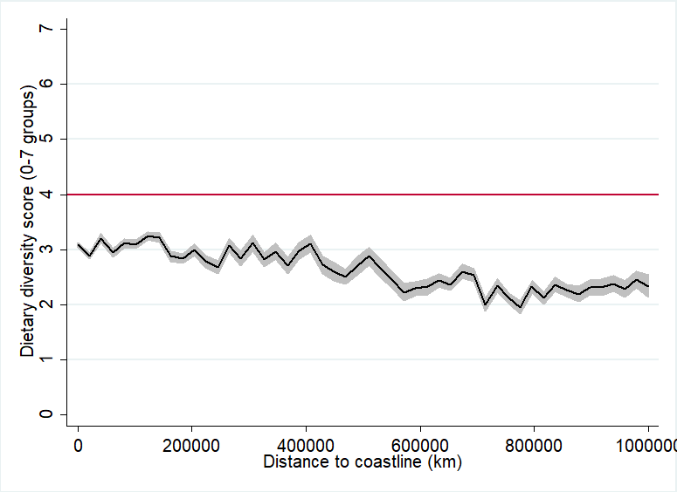

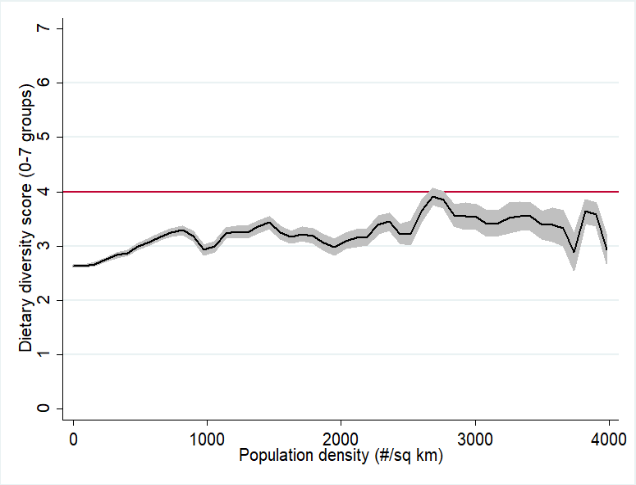

Source: Phase 5 & 6 DHS data for 42 countries. These are local polynomial smoothing estimates with 95% confidence intervals (CI)

**Figure A7. Association of geographic characteristics with household wealth**


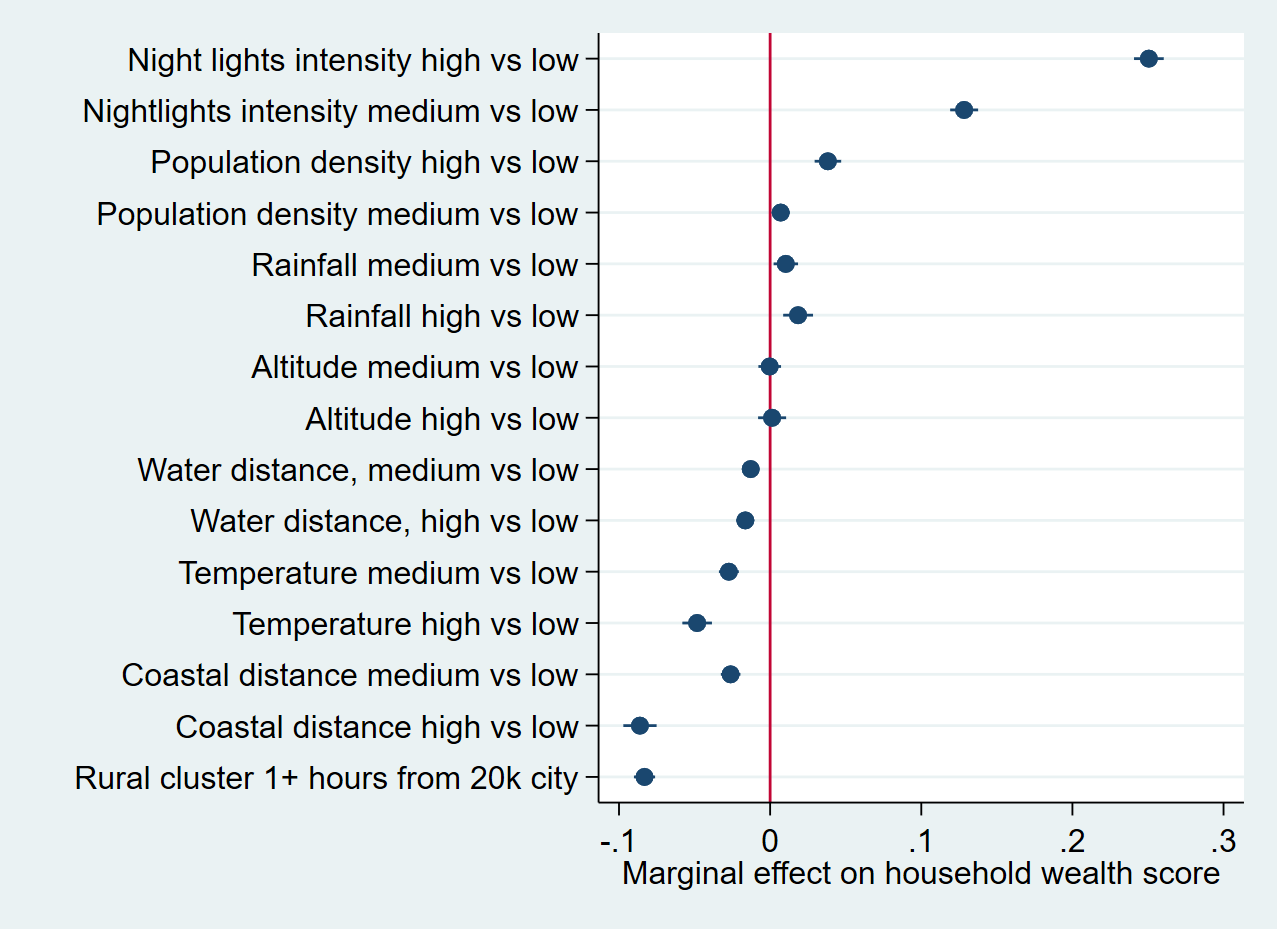


Notes: Data shown are coefficient plots from a regression of the household wealth score, scaled to vary between 0 and 1, on all GIS community variables and country-year fixed effects.

**Table A2. Determinants of minimum dietary diversity (MDD), by age**

|  | (1) | (2) |
| --- | --- | --- |
|  | MDD | MDD |
|  | 6-23 months | 12-23 months |
| Child/household indicators from the DHS |  |  |
| Household wealth, middle vs low | 0.043*** | 0.055*** |
|  | (0.004) | (0.006) |
| Household wealth, high vs low | 0.100*** | 0.123*** |
|  | (0.007) | (0.008) |
| Maternal 1-6 yrs education vs none | 0.026*** | 0.038*** |
|  | (0.005) | (0.006) |
| Maternal 7-9 yrs education vs none | 0.070*** | 0.095*** |
|  | (0.006) | (0.008) |
| Maternal 10+ yrs education vs none | 0.108*** | 0.133*** |
|  | (0.007) | (0.009) |
| Paternal 1-6 yrs education vs none | 0.016*** | 0.022*** |
|  | (0.005) | (0.006) |
| Paternal 7-9 yrs education vs none | 0.026*** | 0.030*** |
|  | (0.006) | (0.008) |
| Paternal 10+ yrs education vs none | 0.042*** | 0.051*** |
|  | (0.006) | (0.008) |
| Health access vs none | 0.074*** | 0.051*** |
|  | (0.004) | (0.005) |
| Child breastfed immediately vs not | 0.013*** | 0.016*** |
|  | (0.004) | (0.004) |
| Mother decides own healthcare vs not | -0.006 | -0.005 |
|  | (0.004) | (0.005) |
| Child is male vs female | 0.001 | -0.001 |
|  | (0.003) | (0.004) |
|  |  |  |
| Geographic characteristics of household locations |  |  |
| Remote location vs not remote | -0.009* | -0.007 |
|  | (0.005) | (0.006) |
| Night lights intensity, middle vs low | 0.012* | 0.017** |
|  | (0.007) | (0.008) |
| Night lights intensity, high vs low | 0.031*** | 0.043*** |
|  | (0.007) | (0.009) |
| Population density, middle vs low | 0.011** | 0.010 |
|  | (0.005) | (0.006) |
| Population density, high vs low | 0.013* | 0.014* |
|  | (0.007) | (0.008) |
| Distance to coastline, middle vs low | 0.014** | 0.023*** |
|  | (0.006) | (0.007) |
| Distance to coastline, high vs low | 0.003 | 0.004 |
|  | (0.008) | (0.011) |
| Distance to water body, middle vs low | 0.009** | 0.011* |
|  | (0.005) | (0.006) |
| Distance to water body, high vs low | 0.007 | 0.005 |
|  | (0.005) | (0.006) |
| Mean rainfall, middle vs low | 0.020*** | 0.026*** |
|  | (0.006) | (0.007) |
| Mean rainfall, high vs low | 0.014** | 0.016* |
|  | (0.007) | (0.009) |
| Mean temperature, middle vs low | -0.030*** | -0.039*** |
|  | (0.006) | (0.008) |
| Mean temperature, high vs low | -0.042*** | -0.039*** |
|  | (0.008) | (0.011) |
| Altitude by cluster, middle vs low | 0.004 | 0.005 |
|  | (0.006) | (0.008) |
| Altitude by cluster, high vs low | -0.002 | 0.002 |
|  | (0.008) | (0.010) |
|  |  |  |
| Age-in-month dummies included? | Yes | Yes |
| Country-year fixed effects included? | Yes | Yes |
|  |  |  |
| R-squared | 0.254 | 0.250 |
| N | 76,641 | 49,123 |

Note: Significance levels shown are estimated using cluster-robust standard errors and are denoted: ***p<0.01, **p<0.05, *p<0.1. Controls included but not reported are child age dummies, country and survey fixed effects.

**Table A3. Robustness of DDS determinants to exclusion of covariates**

|  | (1) | (2) | (3) |
| --- | --- | --- | --- |
|  | Full model | Household variables only | Community variables only |
| Child/household indicators from the DHS |  |  |  |
| Household wealth, middle vs low | 0.171*** | 0.223*** |  |
|  | (0.019) | (0.019) |  |
| Household wealth, high vs low | 0.424*** | 0.548*** |  |
|  | (0.029) | (0.026) |  |
| Maternal 1-6 yrs education vs none | 0.160*** | 0.171*** |  |
|  | (0.021) | (0.021) |  |
| Maternal 7-9 yrs education vs none | 0.342*** | 0.358*** |  |
|  | (0.026) | (0.026) |  |
| Maternal 10+ yrs education vs none | 0.518*** | 0.550*** |  |
|  | (0.029) | (0.029) |  |
| Paternal 1-6 yrs education vs none | 0.113*** | 0.128*** |  |
|  | (0.021) | (0.021) |  |
| Paternal 7-9 yrs education vs none | 0.151*** | 0.168*** |  |
|  | (0.026) | (0.026) |  |
| Paternal 10+ yrs education vs none | 0.207*** | 0.230*** |  |
|  | (0.025) | (0.025) |  |
| Health access vs none | 0.179*** | 0.197*** |  |
|  | (0.017) | (0.017) |  |
| Child breastfed immediately vs not | 0.051*** | 0.055*** |  |
|  | (0.015) | (0.015) |  |
| Mother decides own healthcare vs not | -0.000 | 0.011 |  |
|  | (0.017) | (0.017) |  |
| Child is male vs female | -0.004 | -0.004 |  |
|  | (0.013) | (0.013) |  |
|  | |  |  |
| Geographic characteristics of household locations | |  |  |
| Remote location vs not remote | -0.030 |  | -0.139*** |
|  | (0.023) |  | (0.023) |
| Night lights intensity, middle vs low | 0.056* |  | 0.189*** |
|  | (0.029) |  | (0.030) |
| Night lights intensity, high vs low | 0.180*** |  | 0.459*** |
|  | (0.032) |  | (0.032) |
| Population density, middle vs low | 0.045** |  | 0.059*** |
|  | (0.022) |  | (0.023) |
| Population density, high vs low | 0.054* |  | 0.103*** |
|  | (0.029) |  | (0.030) |
| Distance to coastline, middle vs low | 0.087*** |  | 0.035 |
|  | (0.025) |  | (0.026) |
| Distance to coastline, high vs low | 0.020 |  | -0.161*** |
|  | (0.037) |  | (0.038) |
| Distance to water body, middle vs low | 0.027 |  | 0.014 |
|  | (0.020) |  | (0.021) |
| Distance to water body, high vs low | 0.014 |  | -0.004 |
|  | (0.022) |  | (0.022) |
| Mean rainfall, middle vs low | 0.142*** |  | 0.215*** |
|  | (0.027) |  | (0.028) |
| Mean rainfall, high vs low | 0.111*** |  | 0.251*** |
|  | (0.032) |  | (0.032) |
| Mean temperature, middle vs low | -0.120*** |  | -0.167*** |
|  | (0.026) |  | (0.027) |
| Mean temperature, high vs low | -0.137*** |  | -0.208*** |
|  | (0.036) |  | (0.038) |
| Altitude by cluster, middle vs low | 0.023 |  | 0.010 |
|  | (0.027) |  | (0.028) |
| Altitude by cluster, high vs low | 0.014 |  | 0.015 |
|  | (0.035) |  | (0.036) |
|  |  |  |  |
| Age-in-month dummies included? | Yes | Yes | Yes |
| Country-year fixed effects included? | Yes | Yes | Yes |
|  |  |  |  |
| R-squared | 0.311 | 0.308 | 0.286 |
| N | 49123 | 49123 | 49123 |

Notes: Cluster-robust standard errors were used to estimate significance levels denoted: ***p<0.01, **p<0.05, *p<0.1. Controls included but not reported are child age dummies, country and survey fixed effects.

**Table A4. Determinants of nutrient-rich vegetal food consumption by infants in Africa**

|  | **DGL Veg** | **vA-rich fruit, veg** | **Other fruit, veg** | **Legumes, nuts** |
| --- | --- | --- | --- | --- |
|  |  |  |  |  |
| Child/household indicators from the DHS |  |  |  |  |
| Household wealth, middle vs low | -0.015** | 0.020*** | 0.022*** | -0.003 |
|  | (0.006) | (0.005) | (0.005) | (0.005) |
| Household wealth, high vs low | -0.030*** | 0.043*** | 0.040*** | -0.012 |
|  | (0.009) | (0.009) | (0.008) | (0.009) |
| Maternal 1-6 yrs education vs none | 0.001 | 0.013** | 0.008 | 0.007 |
|  | (0.006) | (0.006) | (0.005) | (0.006) |
| Maternal 7-9 yrs education vs none | 0.017** | 0.020** | 0.030*** | 0.036*** |
|  | (0.009) | (0.008) | (0.007) | (0.008) |
| Maternal 10+ yrs education vs none | 0.009 | 0.029*** | 0.051*** | 0.043*** |
|  | (0.010) | (0.010) | (0.009) | (0.009) |
| Paternal 1-6 yrs education vs none | 0.008 | 0.020*** | 0.019*** | 0.023*** |
|  | (0.006) | (0.006) | (0.005) | (0.006) |
| Paternal 7-9 yrs education vs none | 0.004 | 0.014* | 0.024*** | 0.023*** |
|  | (0.008) | (0.008) | (0.007) | (0.008) |
| Paternal 10+ yrs education vs none | 0.001 | 0.026*** | 0.030*** | 0.026*** |
|  | (0.008) | (0.008) | (0.007) | (0.007) |
| Health access vs none | 0.020*** | 0.030*** | 0.039*** | 0.018*** |
|  | (0.006) | (0.006) | (0.006) | (0.006) |
| Child breastfed immediately vs not | 0.008 | 0.020*** | 0.010** | 0.007 |
|  | (0.005) | (0.005) | (0.004) | (0.005) |
| Mother decides own healthcare vs not | 0.003 | -0.011** | -0.007 | 0.006 |
|  | (0.005) | (0.005) | (0.004) | (0.005) |
| Child is male vs female | 0.001 | -0.002 | 0.004 | 0.001 |
|  | (0.004) | (0.004) | (0.003) | (0.004) |
|  |  |  |  |  |
| Geographic characteristics of household locations | |  |  |  |
| Remote location vs not remote | 0.010 | -0.006 | -0.006 | 0.002 |
|  | (0.007) | (0.007) | (0.006) | (0.007) |
| Night lights intensity, middle vs low | 0.002 | 0.010 | 0.004 | -0.013 |
|  | (0.010) | (0.010) | (0.009) | (0.009) |
| Night lights intensity, high vs low | -0.009 | 0.007 | 0.028*** | 0.015 |
|  | (0.011) | (0.010) | (0.009) | (0.010) |
| Population density, middle vs low | -0.008 | -0.003 | 0.019*** | 0.005 |
|  | (0.007) | (0.007) | (0.005) | (0.007) |
| Population density, high vs low | 0.007 | 0.023** | 0.032*** | 0.009 |
|  | (0.011) | (0.010) | (0.008) | (0.010) |
| Distance to coastline, middle vs low | 0.019* | 0.021* | 0.003 | -0.001 |
|  | (0.010) | (0.011) | (0.009) | (0.010) |
| Distance to coastline, high vs low | 0.123*** | 0.023* | -0.008 | 0.016 |
|  | (0.013) | (0.014) | (0.011) | (0.013) |
| Distance to water body, middle vs low | 0.022*** | 0.017** | 0.011* | 0.018*** |
|  | (0.007) | (0.007) | (0.006) | (0.006) |
| Distance to water body, high vs low | 0.025*** | 0.012* | -0.002 | 0.031*** |
|  | (0.008) | (0.007) | (0.006) | (0.007) |
| Mean rainfall, middle vs low | 0.039*** | 0.022*** | 0.012** | 0.035*** |
|  | (0.009) | (0.007) | (0.006) | (0.007) |
| Mean rainfall, high vs low | 0.065*** | 0.034*** | 0.044*** | 0.039*** |
|  | (0.012) | (0.012) | (0.009) | (0.011) |
| Mean temperature, middle vs low | -0.007 | -0.056*** | -0.029*** | -0.048*** |
|  | (0.010) | (0.010) | (0.009) | (0.010) |
| Mean temperature, high vs low | -0.016 | -0.031** | -0.045*** | -0.063*** |
|  | (0.014) | (0.014) | (0.011) | (0.013) |
| Altitude by cluster, middle vs low | 0.021* | 0.003 | -0.003 | -0.002 |
|  | (0.011) | (0.012) | (0.010) | (0.010) |
| Altitude by cluster, high vs low | 0.060*** | -0.006 | 0.008 | 0.046*** |
|  | (0.016) | (0.016) | (0.013) | (0.014) |
| Age-in-month dummies included? | Yes | Yes | Yes | Yes |
| Country-year fixed effects included? | Yes | Yes | Yes | Yes |
|  |  |  |  |  |
| R-squared | 0.167 | 0.093 | 0.110 | 0.130 |
| N | 42,794 | 42,794 | 42,794 | 42,794 |

Notes: Cluster-robust standard errors were used to estimate significance levels denoted: ***p<0.01, **p<0.05, *p<0.1. Controls included but not reported are child age dummies, country and survey fixed effects.

**Table A5. Determinants of animal-sourced food consumption by infants in Africa**

|  | (1) | (2) | (3) | (4) |
| --- | --- | --- | --- | --- |
|  | **Dairy** | **Eggs** | **Meat, organs** | **Fish^b^** |
|  |  |  |  |  |
| Child/household indicators from the DHS |  |  |  |  |
| Household wealth, middle vs low | 0.041*** | 0.028*** | 0.031*** | 0.011*** |
|  | (0.005) | (0.004) | (0.004) | (0.004) |
| Household wealth, high vs low | 0.131*** | 0.057*** | 0.086*** | -0.000 |
|  | (0.007) | (0.006) | (0.006) | (0.006) |
| Maternal 1-6 yrs education vs none | 0.014*** | 0.016*** | 0.009** | 0.041*** |
|  | (0.005) | (0.004) | (0.004) | (0.005) |
| Maternal 7-9 yrs education vs none | 0.064*** | 0.033*** | 0.036*** | 0.037*** |
|  | (0.006) | (0.005) | (0.006) | (0.006) |
| Maternal 10+ yrs education vs none | 0.114*** | 0.055*** | 0.077*** | 0.034*** |
|  | (0.007) | (0.006) | (0.006) | (0.006) |
| Paternal 1-6 yrs education vs none | -0.005 | 0.015*** | 0.005 | 0.032*** |
|  | (0.005) | (0.004) | (0.005) | (0.005) |
| Paternal 7-9 yrs education vs none | 0.019*** | 0.023*** | 0.021*** | 0.028*** |
|  | (0.006) | (0.005) | (0.006) | (0.006) |
| Paternal 10+ yrs education vs none | 0.028*** | 0.032*** | 0.020*** | 0.043*** |
|  | (0.006) | (0.005) | (0.006) | (0.006) |
| Health access vs none | 0.053*** | 0.052*** | 0.050*** | 0.006 |
|  | (0.004) | (0.004) | (0.004) | (0.004) |
| Child breastfed immediately vs not | -0.006 | 0.017*** | 0.012*** | 0.009*** |
|  | (0.004) | (0.003) | (0.003) | (0.003) |
| Mother decides own healthcare vs not | -0.016*** | 0.001 | -0.003 | 0.006* |
|  | (0.004) | (0.003) | (0.004) | (0.004) |
| Child is male vs female | 0.007** | -0.004 | 0.000 | 0.000 |
|  | (0.003) | (0.003) | (0.003) | (0.003) |
|  |  |  |  |  |
| Geographic characteristics of household locations | |  |  |  |
| Remote location vs not remote | 0.012** | -0.005 | -0.007 | -0.009* |
|  | (0.005) | (0.004) | (0.005) | (0.005) |
| Night lights intensity, middle vs low | 0.027*** | 0.007 | 0.029*** | 0.007 |
|  | (0.007) | (0.006) | (0.006) | (0.006) |
| Night lights intensity, high vs low | 0.069*** | 0.029*** | 0.050*** | -0.001 |
|  | (0.008) | (0.006) | (0.007) | (0.007) |
| Population density, middle vs low | -0.007 | -0.004 | 0.015*** | -0.012** |
|  | (0.005) | (0.004) | (0.005) | (0.005) |
| Population density, high vs low | -0.000 | -0.005 | 0.006 | -0.026*** |
|  | (0.007) | (0.006) | (0.006) | (0.007) |
| Distance to coastline, middle vs low | 0.014** | 0.011** | 0.019*** | -0.013** |
|  | (0.006) | (0.005) | (0.006) | (0.006) |
| Distance to coastline, high vs low | 0.023** | -0.020*** | 0.017** | -0.037*** |
|  | (0.010) | (0.007) | (0.008) | (0.009) |
| Distance to water body, middle vs low | -0.002 | 0.011*** | 0.008* | -0.042*** |
|  | (0.005) | (0.004) | (0.004) | (0.005) |
| Distance to water body, high vs low | -0.004 | 0.010** | 0.015*** | -0.052*** |
|  | (0.005) | (0.004) | (0.005) | (0.005) |
| Mean rainfall, middle vs low | -0.044*** | 0.021*** | 0.022*** | 0.045*** |
|  | (0.007) | (0.005) | (0.006) | (0.006) |
| Mean rainfall, high vs low | -0.064*** | 0.044*** | 0.022*** | 0.099*** |
|  | (0.008) | (0.006) | (0.007) | (0.008) |
| Mean temperature, middle vs low | -0.009 | -0.017*** | -0.001 | 0.025*** |
|  | (0.007) | (0.006) | (0.006) | (0.006) |
| Mean temperature, high vs low | -0.008 | -0.020** | 0.024*** | 0.051*** |
|  | (0.010) | (0.008) | (0.008) | (0.009) |
| Altitude by cluster, middle vs low | -0.009 | 0.021*** | 0.013** | -0.045*** |
|  | (0.006) | (0.006) | (0.006) | (0.007) |
| Altitude by cluster, high vs low | 0.001 | 0.028*** | -0.032*** | -0.057*** |
|  | (0.008) | (0.008) | (0.008) | (0.008) |
| Age-in-month dummies included? | Yes | Yes | Yes | Yes |
| Country-year fixed effects included? | Yes | Yes | Yes | Yes |
|  |  |  |  |  |
| R-squared | 0.276 | 0.187 | 0.232 | 0.163 |
| N | 76,641 | 76,641 | 76,641 | 70,137 |

Notes: Cluster-robust standard errors were used to estimate significance levels denoted: ***p<0.01, **p<0.05, *p<0.1. Controls included but not reported are child age dummies, country and survey fixed effects.

**Table A6. Comparing income elasticities with wealth effects, by food group in Africa**

|  | **Household-level income elasticity (Colen et al. 2018)** | **Child-level middle vs low wealth effect**  **(this study)** | **Child-level high vs low wealth effect**  **(this study)** |
| --- | --- | --- | --- |
| Tubers | 0.32 | 0.022*** | 0.017** |
| Cereals | 0.36 | -0.003 | -0.006 |
| Legumes and nuts | 0.46 | -0.002 | -0.007 |
| Fruits and vegetables | 0.61 | 0.000 | 0.018* |
| DGL vegetables | Not available | -0.025*** | -0.048*** |
| Dairy | 0.81 | 0.032*** | 0.176*** |
| Meat/organs, fish, eggs | 0.80 | 0.058*** | 0.108*** |
| Meat/organs |  | 0.036*** | 0.099*** |
| Eggs |  | 0.021*** | 0.075*** |
| Fish | Not available | 0.019*** | 0.021** |
|  |  |  |  |
| *Correlation with household-level income elasticity* |  | *0.67* | *0.86* |

Notes: The household level income elasticities are the median elasticities for sub-Saharan Africa reported in Appendix Table C2 in Colen et al. (2018). The child-level high vs low wealth effects refers to regressions of child consumption of each food group against DHS child and household level factors, excluding GIS community variables, for sub-Saharan Africa only.

**Table A7. Loading (weights) on the first principal components for the total sample**

| **Asset** | **Total** |
| --- | --- |
| Electricity | 0.45 |
| Radio | 0.12 |
| Television | 0.47 |
| Refrigerator | 0.43 |
| Motorbike | 0.14 |
| Car | 0.24 |
| Floor, basic | 0.38 |
| Improved toilet/water (pipe water, flush toilet) | 0.40 |

**Appendix B**

***Comparisons to previous studies***

We sought to compare our results on child-level demand for food to more conventional economic estimates of household level demand for food. If children are typically fed the foods that the household as a whole is consuming, one would expect these patterns to be similar. One three-country study found that the diets of children and their mothers are very similar (Nguyen et al., 2013), though a study in Bangladesh found that milk was disproportionately fed to young children (Sununtnasuk and Fiedler, 2017). Other more qualitative studies also find that there are often norms that prohibit feeding certain nutritious foods to young children, particularly eggs (Pak-Gorstein et al., 2009).

**Appendix C**

**Table C1. Determinants of dietary diversity scores (DDS), by age**

|  | (1) | (2) |
| --- | --- | --- |
|  | DDS | DDS |
|  | 6-23 months | 12-23 months |
| Child/household indicators from the DHS |  |  |
| Household wealth, middle vs low | 0.146*** | 0.171*** |
|  | (0.016) | (0.019) |
| Household wealth, high vs low | 0.365*** | 0.424*** |
|  | (0.024) | (0.029) |
| Maternal 1-6 yrs education vs none | 0.127*** | 0.160*** |
|  | (0.017) | (0.021) |
| Maternal 7-9 yrs education vs none | 0.284*** | 0.342*** |
|  | (0.021) | (0.026) |
| Maternal 10+ yrs education vs none | 0.448*** | 0.518*** |
|  | (0.024) | (0.029) |
| Paternal 1-6 yrs education vs none | 0.097*** | 0.113*** |
|  | (0.017) | (0.021) |
| Paternal 7-9 yrs education vs none | 0.141*** | 0.151*** |
|  | (0.021) | (0.026) |
| Paternal 10+ yrs education vs none | 0.183*** | 0.207*** |
|  | (0.021) | (0.025) |
| Health access vs none | 0.218*** | 0.179*** |
|  | (0.015) | (0.017) |
| Child breastfed immediately vs not | 0.034*** | 0.051*** |
|  | (0.013) | (0.015) |
| Mother decides own healthcare vs not | 0.006 | -0.000 |
|  | (0.014) | (0.017) |
| Child is male vs female | -0.002 | -0.004 |
|  | (0.011) | (0.013) |
|  |  |  |
| Geographic characteristics of household locations |  |  |
| Remote location vs not remote | -0.031 | -0.030 |
|  | (0.019) | (0.023) |
| Night lights intensity, middle vs low | 0.055** | 0.056* |
|  | (0.024) | (0.029) |
| Night lights intensity, high vs low | 0.143*** | 0.180*** |
|  | (0.027) | (0.032) |
| Population density, middle vs low | 0.045** | 0.045** |
|  | (0.018) | (0.022) |
| Population density, high vs low | 0.048* | 0.054* |
|  | (0.025) | (0.029) |
| Distance to coastline, middle vs low | 0.030 | 0.087*** |
|  | (0.021) | (0.025) |
| Distance to coastline, high vs low | -0.013 | 0.020 |
|  | (0.032) | (0.037) |
| Distance to water body, middle vs low | 0.030* | 0.027 |
|  | (0.017) | (0.020) |
| Distance to water body, high vs low | 0.028 | 0.014 |
|  | (0.018) | (0.022) |
| Mean rainfall, middle vs low | 0.122*** | 0.142*** |
|  | (0.023) | (0.027) |
| Mean rainfall, high vs low | 0.120*** | 0.111*** |
|  | (0.027) | (0.032) |
| Mean temperature, middle vs low | -0.108*** | -0.120*** |
|  | (0.023) | (0.026) |
| Mean temperature, high vs low | -0.153*** | -0.137*** |
|  | (0.031) | (0.036) |
| Altitude by cluster, middle vs low | 0.031 | 0.023 |
|  | (0.023) | (0.027) |
| Altitude by cluster, high vs low | 0.011 | 0.014 |
|  | (0.030) | (0.035) |
|  |  |  |
| Age-in-month dummies included? | Yes | Yes |
| Country-year fixed effects included? | Yes | Yes |
|  |  |  |
| R-squared | 0.357 | 0.311 |
| N | 76,641 | 49,123 |

Note: Significance levels shown are estimated using cluster-robust standard errors and are denoted: ***p<0.01, **p<0.05, *p<0.1. Controls included but not reported are child age dummies, country and survey fixed effects.

**Table C2. Determinants of intake by vegetal food group, for children 6-23 months**

|  | (1) | (2) | (3) | (4) |
| --- | --- | --- | --- | --- |
|  | **DGL Veg** | **vA-rich fruit, veg** | **Other fruit, veg** | **Legumes, nuts** |
|  |  |  |  |  |
| Child/household indicators from the DHS |  |  |  |  |
| Household wealth, middle vs low | -0.016*** | 0.013*** | 0.021*** | 0.016*** |
|  | (0.005) | (0.005) | (0.004) | (0.004) |
| Household wealth, high vs low | -0.009 | 0.056*** | 0.057*** | 0.010 |
|  | (0.006) | (0.007) | (0.006) | (0.006) |
| Maternal 1-6 yrs education vs none | -0.007 | 0.006 | 0.016*** | 0.019*** |
|  | (0.005) | (0.005) | (0.004) | (0.005) |
| Maternal 7-9 yrs education vs none | 0.010 | 0.029*** | 0.047*** | 0.037*** |
|  | (0.006) | (0.006) | (0.006) | (0.006) |
| Maternal 10+ yrs education vs none | 0.029*** | 0.057*** | 0.076*** | 0.034*** |
|  | (0.006) | (0.007) | (0.006) | (0.007) |
| Paternal 1-6 yrs education vs none | 0.006 | 0.007 | 0.005 | 0.029*** |
|  | (0.005) | (0.005) | (0.004) | (0.005) |
| Paternal 7-9 yrs education vs none | 0.008 | 0.017*** | 0.017*** | 0.014** |
|  | (0.006) | (0.006) | (0.006) | (0.006) |
| Paternal 10+ yrs education vs none | 0.014** | 0.024*** | 0.024*** | 0.015*** |
|  | (0.006) | (0.006) | (0.006) | (0.006) |
| Health access vs none | -0.002 | 0.024*** | 0.048*** | 0.012*** |
|  | (0.004) | (0.004) | (0.004) | (0.004) |
| Child breastfed immediately vs not | 0.003 | 0.007** | 0.008** | 0.009** |
|  | (0.004) | (0.004) | (0.003) | (0.004) |
| Mother decides own healthcare vs not | 0.009** | -0.005 | -0.005 | 0.003 |
|  | (0.004) | (0.004) | (0.004) | (0.004) |
| Child is male vs female | 0.000 | -0.000 | 0.002 | -0.001 |
|  | (0.003) | (0.003) | (0.003) | (0.003) |
|  |  |  |  |  |
| Geographic characteristics of household locations | |  |  |  |
| Remote location vs not remote | -0.006 | -0.012** | -0.011** | 0.002 |
|  | (0.005) | (0.005) | (0.005) | (0.005) |
| Night lights intensity, middle vs low | -0.005 | 0.017** | 0.001 | -0.017*** |
|  | (0.007) | (0.007) | (0.006) | (0.006) |
| Night lights intensity, high vs low | 0.000 | 0.009 | 0.016** | -0.005 |
|  | (0.007) | (0.008) | (0.007) | (0.007) |
| Population density, middle vs low | 0.005 | 0.008 | 0.024*** | 0.004 |
|  | (0.005) | (0.005) | (0.005) | (0.005) |
| Population density, high vs low | 0.010 | 0.023*** | 0.026*** | 0.009 |
|  | (0.007) | (0.007) | (0.007) | (0.007) |
| Distance to coastline, middle vs low | 0.004 | 0.006 | -0.012** | 0.010* |
|  | (0.006) | (0.006) | (0.006) | (0.006) |
| Distance to coastline, high vs low | 0.075*** | -0.016* | -0.022*** | 0.007 |
|  | (0.009) | (0.009) | (0.008) | (0.009) |
| Distance to water body, middle vs low | 0.023*** | 0.014*** | 0.011** | 0.007 |
|  | (0.005) | (0.005) | (0.004) | (0.005) |
| Distance to water body, high vs low | 0.029*** | 0.012** | -0.004 | 0.020*** |
|  | (0.005) | (0.005) | (0.005) | (0.005) |
| Mean rainfall, middle vs low | 0.008 | 0.007 | 0.033*** | 0.031*** |
|  | (0.007) | (0.006) | (0.005) | (0.006) |
| Mean rainfall, high vs low | -0.015* | -0.012 | 0.033*** | 0.030*** |
|  | (0.008) | (0.008) | (0.007) | (0.008) |
| Mean temperature, middle vs low | -0.028*** | -0.043*** | -0.009 | -0.020*** |
|  | (0.006) | (0.007) | (0.006) | (0.007) |
| Mean temperature, high vs low | -0.058*** | -0.057*** | -0.030*** | -0.044*** |
|  | (0.009) | (0.009) | (0.009) | (0.009) |
| Altitude by cluster, middle vs low | 0.014** | 0.007 | 0.006 | 0.005 |
|  | (0.006) | (0.007) | (0.006) | (0.006) |
| Altitude by cluster, high vs low | 0.022*** | -0.013 | 0.008 | 0.033*** |
|  | (0.008) | (0.009) | (0.008) | (0.008) |
| Age-in-month dummies included? | Yes | Yes | Yes | Yes |
| Country-year fixed effects included? | Yes | Yes | Yes | Yes |
|  |  |  |  |  |
| R-squared | 0.169 | 0.144 | 0.183 | 0.154 |
| N | 76641 | 76641 | 76641 | 76641 |

Note: As for Table 4.

**Table C3.** **Determinants of intake by animal-sourced food group, for children 6-23 months**

|  | (1) | (2) | (3) | (4) |
| --- | --- | --- | --- | --- |
|  | **Dairy** | **Eggs** | **Meat, organs** | **Fish^b^** |
|  |  |  |  |  |
| Child/household indicators from the DHS |  |  |  |  |
| Household wealth, middle vs low | 0.041*** | 0.028*** | 0.031*** | 0.011*** |
|  | (0.005) | (0.004) | (0.004) | (0.004) |
| Household wealth, high vs low | 0.131*** | 0.057*** | 0.086*** | -0.000 |
|  | (0.007) | (0.006) | (0.006) | (0.006) |
| Maternal 1-6 yrs education vs none | 0.014*** | 0.016*** | 0.009** | 0.041*** |
|  | (0.005) | (0.004) | (0.004) | (0.005) |
| Maternal 7-9 yrs education vs none | 0.064*** | 0.033*** | 0.036*** | 0.037*** |
|  | (0.006) | (0.005) | (0.006) | (0.006) |
| Maternal 10+ yrs education vs none | 0.114*** | 0.055*** | 0.077*** | 0.034*** |
|  | (0.007) | (0.006) | (0.006) | (0.006) |
| Paternal 1-6 yrs education vs none | -0.005 | 0.015*** | 0.005 | 0.032*** |
|  | (0.005) | (0.004) | (0.005) | (0.005) |
| Paternal 7-9 yrs education vs none | 0.019*** | 0.023*** | 0.021*** | 0.028*** |
|  | (0.006) | (0.005) | (0.006) | (0.006) |
| Paternal 10+ yrs education vs none | 0.028*** | 0.032*** | 0.020*** | 0.043*** |
|  | (0.006) | (0.005) | (0.006) | (0.006) |
| Health access vs none | 0.053*** | 0.052*** | 0.050*** | 0.006 |
|  | (0.004) | (0.004) | (0.004) | (0.004) |
| Child breastfed immediately vs not | -0.006 | 0.017*** | 0.012*** | 0.009*** |
|  | (0.004) | (0.003) | (0.003) | (0.003) |
| Mother decides own healthcare vs not | -0.016*** | 0.001 | -0.003 | 0.006* |
|  | (0.004) | (0.003) | (0.004) | (0.004) |
| Child is male vs female | 0.007** | -0.004 | 0.000 | 0.000 |
|  | (0.003) | (0.003) | (0.003) | (0.003) |
|  |  |  |  |  |
| Geographic characteristics of household locations | |  |  |  |
| Remote location vs not remote | 0.012** | -0.005 | -0.007 | -0.009* |
|  | (0.005) | (0.004) | (0.005) | (0.005) |
| Night lights intensity, middle vs low | 0.027*** | 0.007 | 0.029*** | 0.007 |
|  | (0.007) | (0.006) | (0.006) | (0.006) |
| Night lights intensity, high vs low | 0.069*** | 0.029*** | 0.050*** | -0.001 |
|  | (0.008) | (0.006) | (0.007) | (0.007) |
| Population density, middle vs low | -0.007 | -0.004 | 0.015*** | -0.012** |
|  | (0.005) | (0.004) | (0.005) | (0.005) |
| Population density, high vs low | -0.000 | -0.005 | 0.006 | -0.026*** |
|  | (0.007) | (0.006) | (0.006) | (0.007) |
| Distance to coastline, middle vs low | 0.014** | 0.011** | 0.019*** | -0.013** |
|  | (0.006) | (0.005) | (0.006) | (0.006) |
| Distance to coastline, high vs low | 0.023** | -0.020*** | 0.017** | -0.037*** |
|  | (0.010) | (0.007) | (0.008) | (0.009) |
| Distance to water body, middle vs low | -0.002 | 0.011*** | 0.008* | -0.042*** |
|  | (0.005) | (0.004) | (0.004) | (0.005) |
| Distance to water body, high vs low | -0.004 | 0.010** | 0.015*** | -0.052*** |
|  | (0.005) | (0.004) | (0.005) | (0.005) |
| Mean rainfall, middle vs low | -0.044*** | 0.021*** | 0.022*** | 0.045*** |
|  | (0.007) | (0.005) | (0.006) | (0.006) |
| Mean rainfall, high vs low | -0.064*** | 0.044*** | 0.022*** | 0.099*** |
|  | (0.008) | (0.006) | (0.007) | (0.008) |
| Mean temperature, middle vs low | -0.009 | -0.017*** | -0.001 | 0.025*** |
|  | (0.007) | (0.006) | (0.006) | (0.006) |
| Mean temperature, high vs low | -0.008 | -0.020** | 0.024*** | 0.051*** |
|  | (0.010) | (0.008) | (0.008) | (0.009) |
| Altitude by cluster, middle vs low | -0.009 | 0.021*** | 0.013** | -0.045*** |
|  | (0.006) | (0.006) | (0.006) | (0.007) |
| Altitude by cluster, high vs low | 0.001 | 0.028*** | -0.032*** | -0.057*** |
|  | (0.008) | (0.008) | (0.008) | (0.008) |
| Age-in-month dummies included? | Yes | Yes | Yes | Yes |
| Country-year fixed effects included? | Yes | Yes | Yes | Yes |
|  |  |  |  |  |
| R-squared | 0.169 | 0.144 | 0.183 | 0.154 |
| N | 76641 | 76641 | 76641 | 76641 |

Notes: Cluster-robust standard errors were used to estimate significance levels denoted: ***p<0.01, **p<0.05, *p<0.1. Controls included but not reported are child age dummies, country and survey fixed effects

**Table C4. Determinants of intake by animal-sourced food group, for children 6-23 months**

|  | (1) | (2) | (3) | (4) |
| --- | --- | --- | --- | --- |
|  | **Dairy** | **Eggs** | **Meat, organs** | **Fish^b^** |
|  |  |  |  |  |
| Child/household indicators from the DHS |  |  |  |  |
| Household wealth, middle vs low | 0.041*** | 0.028*** | 0.031*** | 0.011*** |
|  | (0.005) | (0.004) | (0.004) | (0.004) |
| Household wealth, high vs low | 0.131*** | 0.057*** | 0.086*** | -0.000 |
|  | (0.007) | (0.006) | (0.006) | (0.006) |
| Maternal 1-6 yrs education vs none | 0.014*** | 0.016*** | 0.009** | 0.041*** |
|  | (0.005) | (0.004) | (0.004) | (0.005) |
| Maternal 7-9 yrs education vs none | 0.064*** | 0.033*** | 0.036*** | 0.037*** |
|  | (0.006) | (0.005) | (0.006) | (0.006) |
| Maternal 10+ yrs education vs none | 0.114*** | 0.055*** | 0.077*** | 0.034*** |
|  | (0.007) | (0.006) | (0.006) | (0.006) |
| Paternal 1-6 yrs education vs none | -0.005 | 0.015*** | 0.005 | 0.032*** |
|  | (0.005) | (0.004) | (0.005) | (0.005) |
| Paternal 7-9 yrs education vs none | 0.019*** | 0.023*** | 0.021*** | 0.028*** |
|  | (0.006) | (0.005) | (0.006) | (0.006) |
| Paternal 10+ yrs education vs none | 0.028*** | 0.032*** | 0.020*** | 0.043*** |
|  | (0.006) | (0.005) | (0.006) | (0.006) |
| Health access vs none | 0.053*** | 0.052*** | 0.050*** | 0.006 |
|  | (0.004) | (0.004) | (0.004) | (0.004) |
| Child breastfed immediately vs not | -0.006 | 0.017*** | 0.012*** | 0.009*** |
|  | (0.004) | (0.003) | (0.003) | (0.003) |
| Mother decides own healthcare vs not | -0.016*** | 0.001 | -0.003 | 0.006* |
|  | (0.004) | (0.003) | (0.004) | (0.004) |
| Child is male vs female | 0.007** | -0.004 | 0.000 | 0.000 |
|  | (0.003) | (0.003) | (0.003) | (0.003) |
|  |  |  |  |  |
| Geographic characteristics of household locations | |  |  |  |
| Remote location vs not remote | 0.012** | -0.005 | -0.007 | -0.009* |
|  | (0.005) | (0.004) | (0.005) | (0.005) |
| Night lights intensity, middle vs low | 0.027*** | 0.007 | 0.029*** | 0.007 |
|  | (0.007) | (0.006) | (0.006) | (0.006) |
| Night lights intensity, high vs low | 0.069*** | 0.029*** | 0.050*** | -0.001 |
|  | (0.008) | (0.006) | (0.007) | (0.007) |
| Population density, middle vs low | -0.007 | -0.004 | 0.015*** | -0.012** |
|  | (0.005) | (0.004) | (0.005) | (0.005) |
| Population density, high vs low | -0.000 | -0.005 | 0.006 | -0.026*** |
|  | (0.007) | (0.006) | (0.006) | (0.007) |
| Distance to coastline, middle vs low | 0.014** | 0.011** | 0.019*** | -0.013** |
|  | (0.006) | (0.005) | (0.006) | (0.006) |
| Distance to coastline, high vs low | 0.023** | -0.020*** | 0.017** | -0.037*** |
|  | (0.010) | (0.007) | (0.008) | (0.009) |
| Distance to water body, middle vs low | -0.002 | 0.011*** | 0.008* | -0.042*** |
|  | (0.005) | (0.004) | (0.004) | (0.005) |
| Distance to water body, high vs low | -0.004 | 0.010** | 0.015*** | -0.052*** |
|  | (0.005) | (0.004) | (0.005) | (0.005) |
| Mean rainfall, middle vs low | -0.044*** | 0.021*** | 0.022*** | 0.045*** |
|  | (0.007) | (0.005) | (0.006) | (0.006) |
| Mean rainfall, high vs low | -0.064*** | 0.044*** | 0.022*** | 0.099*** |
|  | (0.008) | (0.006) | (0.007) | (0.008) |
| Mean temperature, middle vs low | -0.009 | -0.017*** | -0.001 | 0.025*** |
|  | (0.007) | (0.006) | (0.006) | (0.006) |
| Mean temperature, high vs low | -0.008 | -0.020** | 0.024*** | 0.051*** |
|  | (0.010) | (0.008) | (0.008) | (0.009) |
| Altitude by cluster, middle vs low | -0.009 | 0.021*** | 0.013** | -0.045*** |
|  | (0.006) | (0.006) | (0.006) | (0.007) |
| Altitude by cluster, high vs low | 0.001 | 0.028*** | -0.032*** | -0.057*** |
|  | (0.008) | (0.008) | (0.008) | (0.008) |
| Age-in-month dummies included? | Yes | Yes | Yes | Yes |
| Country-year fixed effects included? | Yes | Yes | Yes | Yes |
|  |  |  |  |  |
| R-squared | 0.169 | 0.144 | 0.183 | 0.154 |
| N | 76641 | 76641 | 76641 | 76641 |

Notes: Cluster-robust standard errors were used to estimate significance levels denoted: ***p<0.01, **p<0.05, *p<0.1. Controls included but not reported are child age dummies, country and survey fixed effects

**Table C5. Heterogeneity in determinants of dietary diversity scores, for children 6-23 months by region**

**Panel A: Child/household indicators**

|  | (1) | (2) | (3) | (4) | (5) |
| --- | --- | --- | --- | --- | --- |
|  | Sub-Saharan Africa | Asia | Latin America & Caribbean | Middle East & North Africa | East Europe & Central Asia |
|  |  |  |  |  |  |
| Child/household indicators from the DHS |  |  |  |  |  |
| Middle wealth vs low | 0.032*** | 0.052*** | 0.077*** | -0.075 | 0.065 |
|  | (0.005) | (0.012) | (0.012) | (0.126) | (0.125) |
| Upper wealth vs low | 0.082*** | 0.092*** | 0.147*** | 0.052 | 0.060 |
|  | (0.009) | (0.023) | (0.014) | (0.123) | (0.124) |
| Maternal primary education | 0.011** | 0.043*** | 0.090*** | 0.057** | 0.066 |
|  | (0.005) | (0.013) | (0.016) | (0.027) | (0.093) |
| Maternal secondary education | 0.052*** | 0.065*** | 0.151*** | 0.072** | 0.027 |
|  | (0.008) | (0.016) | (0.018) | (0.028) | (0.088) |
| Maternal tertiary education | 0.091*** | 0.101*** | 0.176*** | 0.132*** | 0.042 |
|  | (0.010) | (0.021) | (0.018) | (0.023) | (0.085) |
| Paternal primary education | 0.013** | 0.033** | 0.018 | 0.019 | 0.008 |
|  | (0.005) | (0.013) | (0.016) | (0.028) | (0.129) |
| Paternal secondary education | 0.021*** | 0.059*** | 0.022 | 0.006 | -0.019 |
|  | (0.007) | (0.015) | (0.019) | (0.029) | (0.113) |
| Paternal tertiary education | 0.043*** | 0.100*** | 0.020 | 0.044* | -0.004 |
|  | (0.007) | (0.018) | (0.018) | (0.026) | (0.111) |
| Health access | 0.061*** | 0.112*** | 0.057*** | 0.026 | 0.059*** |
|  | (0.006) | (0.017) | (0.009) | (0.016) | (0.021) |
| Child was breastfed immediately | 0.016*** | 0.011 | 0.007 | 0.008 | 0.023 |
|  | (0.005) | (0.012) | (0.007) | (0.013) | (0.022) |
| Mother can decide on own healthcare | -0.012** | 0.026** | 0.010 | -0.026 | -0.015 |
|  | (0.005) | (0.012) | (0.009) | (0.018) | (0.021) |
| Child is male | -0.003 | 0.008 | 0.007 | -0.002 | -0.017 |
|  | (0.004) | (0.009) | (0.007) | (0.012) | (0.017) |

**Panel B: Geographic characteristics**

|  | Sub-Saharan Africa | Asia | Latin America & Caribbean | Middle East & North Africa | East Europe & Central Asia |
| --- | --- | --- | --- | --- | --- |
|  |  |  |  |  |  |
| Geographic characteristics of household locations | |  |  |  |  |
| Remote location vs not remote | -0.006 | -0.031** | -0.016 | 0.013 | 0.003 |
|  | (0.006) | (0.015) | (0.012) | (0.024) | (0.029) |
| Night lights intensity, middle vs low | 0.009 | -0.004 | 0.005 | 0.088 | 0.024 |
|  | (0.009) | (0.016) | (0.013) | (0.077) | (0.034) |
| Night lights intensity, high vs low | 0.048*** | 0.014 | -0.017 | 0.176** | 0.042 |
|  | (0.010) | (0.023) | (0.015) | (0.075) | (0.040) |
| Population density, middle vs low | 0.002 | 0.026 | 0.039*** | 0.021 | -0.009 |
|  | (0.006) | (0.019) | (0.011) | (0.027) | (0.030) |
| Population density, high vs low | 0.011 | 0.023 | 0.041*** | 0.005 | -0.017 |
|  | (0.009) | (0.028) | (0.013) | (0.027) | (0.040) |
| Distance to coastline, middle vs low | 0.033*** | -0.014 | 0.034*** | -0.044*** |  |
|  | (0.010) | (0.016) | (0.011) | (0.016) |  |
| Distance to coastline, high vs low | 0.034*** | -0.035 | -0.043** |  |  |
|  | (0.012) | (0.110) | (0.019) |  |  |
| Distance to water body, middle vs low | 0.009 | 0.026* | -0.001 | 0.014 | -0.036 |
|  | (0.006) | (0.015) | (0.010) | (0.018) | (0.024) |
| Distance to water body, high vs low | 0.005 | 0.003 | -0.005 | 0.073*** | -0.048 |
|  | (0.006) | (0.021) | (0.010) | (0.025) | (0.034) |
| Mean rainfall, middle vs low | 0.022*** | 0.087 | 0.018 |  | -0.069 |
|  | (0.007) | (0.055) | (0.016) |  | (0.079) |
| Mean rainfall, high vs low | 0.055*** | 0.041 | -0.006 |  |  |
|  | (0.010) | (0.055) | (0.016) |  |  |
| Mean temperature, middle vs low | -0.038*** | -0.059** | -0.009 | 0.023 |  |
|  | (0.009) | (0.026) | (0.012) | (0.024) |  |
| Mean temperature, high vs low | -0.022* | -0.039 | -0.051*** | -0.012 |  |
|  | (0.012) | (0.035) | (0.015) | (0.057) |  |
| Altitude by cluster, middle vs low | -0.005 | -0.009 | 0.005 | 0.046 | -0.074 |
|  | (0.010) | (0.022) | (0.010) | (0.033) | (0.049) |
| Altitude by cluster, high vs low | 0.025* | -0.050** | -0.039*** | 0.067* | -0.112** |
|  | (0.014) | (0.025) | (0.014) | (0.034) | (0.053) |
|  |  |  |  |  |  |
| Age-in-month dummies included? | Yes | Yes | Yes | Yes | Yes |
| Country-year fixed effects? | Yes | Yes | Yes | Yes | Yes |
| R-squared | 0.165 | 0.131 | 0.204 | 0.114 | 0.099 |
| N | 42794 | 7968 | 17658 | 5356 | 2865 |

Notes: Cluster-robust standard errors were used to estimate significance levels denoted: ***p<0.01, **p<0.05, *p<0.1. Controls included but not reported are child age dummies, country and survey fixed effects.
